# Development and Evaluation of an Operative Case Length Prediction Model in Adult Surgical Patients

**DOI:** 10.1097/AS9.0000000000000652

**Published:** 2026-02-09

**Authors:** Jacob Walker Rosenthal, Isaac J. Perron, Drew W. Goldberg, Armaan A. Nallicheri, Charles T. Bradford, Charles C. Horn, Kaley Piersanti, Bhavana Kunisetty, John H. Keogh, Gary E. Weissman, Rachel R. Kelz

**Affiliations:** From the * Department of Surgery, Perelman School of Medicine, University of Pennsylvania, Philadelphia, PA; † Core for Precision Resource Utilization, Center for Healthcare Transformation and Innovation, University of Pennsylvania Health System, Philadelphia, PA; ‡ Perioperative and Procedural Services, Hospital of the University of Pennsylvania, Philadelphia, PA; § Pulmonary, Allergy, Critical Care Division, Department of Medicine, Perelman School of Medicine, University of Pennsylvania, Philadelphia, PA.

**Keywords:** Machine learning, Surgical case duration, Operating room efficiency, Predictive modeling, Electronic health records

## Abstract

**Objective:**

To develop a machine learning model that predicts surgical case length and benchmark its performance against an embedded electronic health record (EHR) model.

**Background:**

Surgical care accounts for one-third of U.S. healthcare expenditure. Current case length prediction models are generally overly simplistic and inaccurate or too specialized to have a broad impact, contributing to operating room (OR) inefficiency and dissatisfaction for patients and providers.

**Methods:**

Retrospective analysis of 55,495 surgical cases performed by 299 surgeons between January 2022 and April 2024 at a metropolitan, quaternary care hospital. The dataset was split temporally for training (46,767 cases) and holdout validation (8728 cases). Three separate machine learning models predicted preprocedure, operative, and postprocedure times using patient and provider characteristics, operation details, and hospital features available at least 1 day before surgery. Approximately 22% of cases lacked historical time averages and relied on procedural time heuristics.

**Results:**

The machine learning model significantly outperformed the embedded EHR model, achieving lower root mean squared error (61.0 vs 91.0 minutes; *P* < 0.01), lower mean average error (39.6 vs 51.8 minutes; *P* < 0.01), and higher *R*^2^ (0.78 vs 0.50; *P* < 0.01). The model predicted 213 more cases within ±30 minutes of actual duration. In cases without historical time averages, the model increased cases within ±30 minutes of actual duration (35% vs 29%; *P* < 0.01).

**Conclusions:**

A machine learning model leveraging comprehensive preoperative data significantly improved surgical case length prediction compared to an embedded EHR model. Future implementation has the potential to improve OR efficiency and patient and provider satisfaction.

## INTRODUCTION

In 2023, U.S. healthcare expenditure reached $4.9 trillion and is expected to grow at 5.6% annually through 2032, outpacing projected gross domestic product growth.^1^ Surgical care accounts for roughly one-third of U.S. healthcare spending.^2^ Within surgical care, the operating room (OR) requires costly resources including staff, equipment, and medications. While no generalizable benchmarks exist, 1 cross-sectional study estimated the mean cost of OR time at $36–$37 per minute,^3^ highlighting the potential savings opportunity from reducing idle and over time. The optimization of OR scheduling thus represents a significant cost-reduction opportunity for hospitals and is a key concern for surgeons and their patients, perioperative staff, and hospital administrators.

Perioperative teams rely on case length estimations to best schedule OR block time. There is no standardized process for OR scheduling, and these estimations are obtained through a variety of ways, ranging from the judgment of surgeons and their scheduling assistants to historical case length data embedded in the electronic health record (EHR).^4,5^ Manually adjusting cases introduces variability across providers and their experience, while simple historical case averages do not consider individualized patient and surgeon factors and are not useful for rare or new cases to the hospital. These case estimate inaccuracies can delay OR start times, resulting in longer OR days.^6^ Furthermore, unpredictable OR days place undue burden on surgeons, staff, and perioperative managers, often requiring teams of experienced personnel to navigate daily scheduling challenges.^7^ Altogether, this inefficiency contributes to waste, excess costs, and dissatisfaction for patients and providers.^8^ Most OR scheduling models described in the literature are either too simplistic, lacking in the personalization needed to ensure efficiency, or focused on a single specialty or current procedural terminology (CPT) code, which greatly reduces their scalability and impact.^9–13^

In this study, we built a maching learning(ML) model to predict surgical case length across different service lines with variable case complexity and benchmark the ML model performance against the embedded EHR model and actual case lengths. We hypothesized that the ML model would significantly improve case length prediction compared to an embedded EHR model, especially for cases without historical time averages.

## METHODS

### Study Setting and Data Sources

Surgical data was pulled from Epic Clarity and Caboodle databases, hosted on Azure Databricks. Social Determinants of Health were retrieved from the 2020 zip code tabulation area.^14^ This study was deemed to meet the criteria for exemption, category 4, by the University of Pennsylvania’s Institutional Review Board (Protocol #858502). The manuscript preparation followed the “Transparent Reporting of a Multivariate Prediction Model for Individual Prognosis or Diagnosis” guidelines when applicable.^15^ Code developed for this model is not publically available.

### Participants

All operative procedures at the Hospital of the University of Pennsylvania campus between January 1, 2022 and April 30, 2024, were eligible for inclusion. Procedures were excluded if performed by gastroenterologists, pulmonologists, and radiation oncologists; canceled or otherwise incomplete cases; or flagged with “illogical” time stamps. Illogical timestamps result from human error, as they are tracked when OR personnel click a button or log the time after an event occurred; approximately 6% of cases were removed for this reason (see Supplemental Methods, https://links.lww.com/AOSO/A576). The final dataset was then split temporally, with data from January 1, 2022 to December 31, 2023, used for model training (n = 46,767), and data from January 1, 2024 to April 30, 2024, used for holdout validation (n = 8728); see Supplemental Figure 1, https://links.lww.com/AOSO/A576. Data is from our health system and is not publicly available.

### Feature Development

A full list of model features, their descriptions, and summary statistics can be found in Supplemental Tables 1 and 5, https://links.lww.com/AOSO/A576. Only data known at least 1 day before the surgery date were included in the feature set. This includes: (1) patient demographic factors; for example, age, sex, and race; (2) patient health factors; for example, body mass index (BMI), smoking status, recent and current hospital utilization, active primary care provider on file, history of anesthesia complications, and allergies; (3) patient comorbidities; for example, Elixhauser morbidity index,^16^ claims frailty index,^17^ and multimorbidity^18^; (4) surgery factors, for example, elective or emergent, preoperative diagnosis, anticipated anesthesia method, number of procedures, and number of panels (a panel is a part of the surgery with a distinct procedure); (5) provider factors; for example, running 2 rooms, recent 90-day surgical volume and associated relative value units (RVUs), and individual surgeon (IDs) with >100 cases in training data; and (6) health-system factors; for example, surgery year, surgery day of week, hospital building, OR.

The embedded EHR model relies primarily on minimum sample size averages of historical case times for given criteria combinations related to procedure, provider, and location. EHR model estimates are available before surgery and for ongoing historical reference, but we chose not to include this as an ML model input to ensure any alterations to the EHR model would not impact these predictions. Instead, our approach implemented a custom method to calculate the average historical times that is similar to the EHR approach. First, the scheduled CPT codes for each case were matched to all other cases in the prior 2 years with an identical combination of CPT codes across panels. The order of CPT code entry, panel designation, and surgeon assignment are each considered when using the historical data. As such, 2 like cases may appear procedurally distinct if 1 case lists CPT code 1 in the first position and the other lists the same CPT code in the second position. We then employed a hierarchical approach prioritizing matched samples in this order: (1) hospital, surgeon, and procedures; (2) surgeon and procedures; (3) hospital and procedures; and (4) procedures only. If the sample contained historical times from at least 5 unique cases, the average times of that hierarchy were used (up to 100 most recent cases averaged). If the sample did not contain at least 5 cases, then the hierarchical level below was considered. If there was not sufficient historical volume across all hierarchies for the set of procedure codes, then CPT time heuristics were aggregated. These heuristic times are assigned to each individual CPT code by our system’s perioperative staff, and a simple sum of the scheduled procedures was calculated; possible procedures were given a time value of 0 minutes. Approximately 22% of cases did not have any historical case time estimates and therefore relied on these aggregated heuristics. Categorical variables were either 1-hot encoded or multilabel encoded and given integer values. Continuous features were coerced to float or integer, and imputed missing values with the median.

### Model Development

The primary outcome was patient in-room to out-room time in minutes. In line with previous approaches,^12^ we split the task into 3 separate models, each with the same input features but different targets—(1) preprocedure time (in-room to procedure start); (2) operative time (procedure start to procedure end); and (3) postprocedure time (procedure end to out-room). While we report performance metrics of each individual model, all comparisons to the EHR model use the aggregate predictions across all 3 models to estimate in-room to out-room time. See Supplemental Figure 2, https://links.lww.com/AOSO/A576, for the model development diagram.

Model selection was done by minimizing the root mean squared error (RMSE), although we also report mean absolute error (MAE) and *R*^2^ where appropriate. RSME was chosen as the metric to minimize the model to reduce the frequency of the high magnitude errors. AutoGluOn was initially run to rapidly test a variety of modeling frameworks.^19^ We chose Light Gradient Boosting Machine (LightGBM; version 4.3.0) for all models because it showed comparable error rates to the best, most complex ensemble model, but with considerably shorter fit time.^20^ A full grid search was conducted across various hyperparameters for a model, with 5-times repeated, 10-fold cross-validation used to establish reliable performance estimates. For each fold, model performance on the training subset (‘CV train’) and the held-out testing subset (‘CV test’) was recorded. Metrics were averaged across all 50 iterations, and the best hyperparameters were chosen based on the lowest RMSE, similar RMSE between training and testing data, and the least complex model. There is no recalibration done for this model. The model was run without race as a feature, and there was not a statistically significant difference in the results. See Supplemental Methods, https://links.lww.com/AOSO/A576.

### Model Evaluation

The final model was refit on the entire training data and was used to predict case duration on the cases in 4 months of holdout validation data; we include a comparison to the EHR model as baseline hereafter. We calculated bootstrap accelerated confidence intervals (1 standard deviation) for all metrics.^21^ We assessed model performance by comparing predicted probabilities to observed outcomes using calibration plots for the ML model and the EHR estimates. Last, to assess the clinical impact of our model, we stratified cases based on the magnitude of prediction errors exceeding ±30 and ±60 minutes to estimate the number of inaccurate cases and the total error saved by our model. The MAE is calculated for each case for both models by subtracting the actual case duration from each model estimate. Then for each model, the case is placed into 1 of 5 buckets: (1) severe overestimation, where model predicts greater than +60 minutes over actual; (2) overestimation, +30 to +60 minutes over; (3) accurate, between –30 and +30 minutes; (4) underestimation, −30 to −60 minutes under; and (5) severe underestimation, where model is −60 minutes under or less. We report the number and percent of cases in each bucket, and the total and mean error in these buckets. Calibration curves were also constructed for elective and emergent/urgent cases separately.

### Statistical Analysis

All analyses were conducted on Azure Databricks using Python (v3.10), sci-kit learn (v1.5.0), scipy (v1.11.4), and statsmodels (v0.14.4). Model comparisons of aggregate error were conducted using the nonparametric Wilcoxon signed-ranked test. Comparisons of proportions of estimates from each model that fell into error magnitude groups were conducted using McNemar’s test. There is no class imbalance since this is a regression, but the training cross-validation was stratified by service to ensure those were balanced during model training. Results are considered statistically significant at *P* < 0.05, unless otherwise indicated.

## RESULTS

We analyzed a total of 55,495 cases that were performed by 299 surgeons. The training set included 46,767 cases, and the holdout set included 8728 cases. In the full cohort (N = 55,495), the median patient age (IQR) was 60.1 years (45.1–69.9) (Table 1). In the training set, urology (n = 6136, 13%), otolaryngology (n = 5939, 13%), and surgical oncology (n = 5178, 11%) had the highest procedural volumes. Case distributions across surgical specialties are summarized in Supplemental Table 5, https://links.lww.com/AOSO/A576. Most procedures were booked as elective (93%, n = 43,518), with 52% (n = 28,857) performed in the ambulatory setting. The median total actual in-room to out-room time across all included surgical procedures was 142 minutes (IQR, 89–226). The median preprocedure time was 39 minutes (IQR, 30–55). The median operative time was 83 minutes (IQR, 41–148), and the median postprocedure time was 17 minutes (IQR, 14–22) (Supplemental Table 6, https://links.lww.com/AOSO/A576). The subgroup without historical data comprises 11,936 (22%) of cases.

**TABLE 1.** Model Feature Summary Statistics

Variable	Total, n (%)	Training Set, n (%)	Holdout Set, n (%)
Total (n)	55,495 (100)	46,767 (100)	8728 (100)
Age, median (IQI)	60.1 (45.1–70)	60.1 (45.1–69.9)	60.9 (45.3–70.4)
English speaking, yes	53,565 (96.5)	45,163 (96.6)	8402 (96.3)
Marital status, married	31,469 (56.7)	26,496 (56.7)	4973 (57.0)
Active PCP	47,586 (85.7)	40,098 (85.7)	7488 (85.8)
Active MyChart	47,799 (86.1)	39,931 (85.4)	7868 (90.1)
Female sex	26,835 (48.4)	22,585 (48.3)	4250 (48.7)
Multimorbid	11,033 (19.9)	9170 (19.6)	1863 (21.3)
Anesthesia complexity	7745 (14)	6498 (13.9)	1247 (14.3)
Previous emergency encounter	10,341 (18.6)	8607 (18.4)	1734 (19.9)
Previous inpatient encounter	12,753 (23)	10,737 (23.0)	2016 (23.1)
Payer			
Managed	33,886 (61.1)	28,316 (60.6)	5570 (63.8)
Medicare	21,149 (38.1)	17,699 (37.9)	3450 (39.5)
Medicaid	6229 (11.2)	5320 (11.4)	909 (10.4)
Self-pay	889 (1.6)	758 (1.6)	131 (1.5)
Race			
White	37,361 (67.3)	31,533 (67.4)	5828 (66.8)
Black	11,816 (21.3)	9947 (21.3)	1869 (21.4)
Asian	1997 (3.6)	1665 (3.6)	332 (3.8)
Hispanic	2176 (3.9)	1854 (4.0)	322 (3.7)
Multiple	675 (1.2)	556 (1.2)	119 (1.4)
Social determinates of health			
Health literacy risk	1204 (2.2)	849 (1.8)	355 (4.1)
Housing risk	973 (1.8)	748 (1.6)	225 (2.6)
Social isolation risk	88 (0.2)	65 (0.1)	23 (0.3)

### Model Development and Performance

**FIGURE 1.**
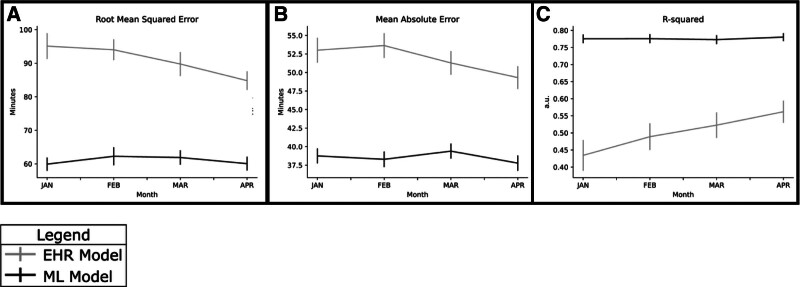
Performance comparison between the ML model and the EHR model in the holdout set by month. RSME (A), MAE (B), and *R*^2^ (C) were calculated monthly from January to April to assess predictive accuracy. The ML model consistently outperformed the EHR model across all metrics, demonstrating lower RMSE and MAE values and higher *R*^2^ values each month. Error bars represent ± 1 standard deviation. These findings indicate superior accuracy and stability of the ML model over time.

**FIGURE 2.**
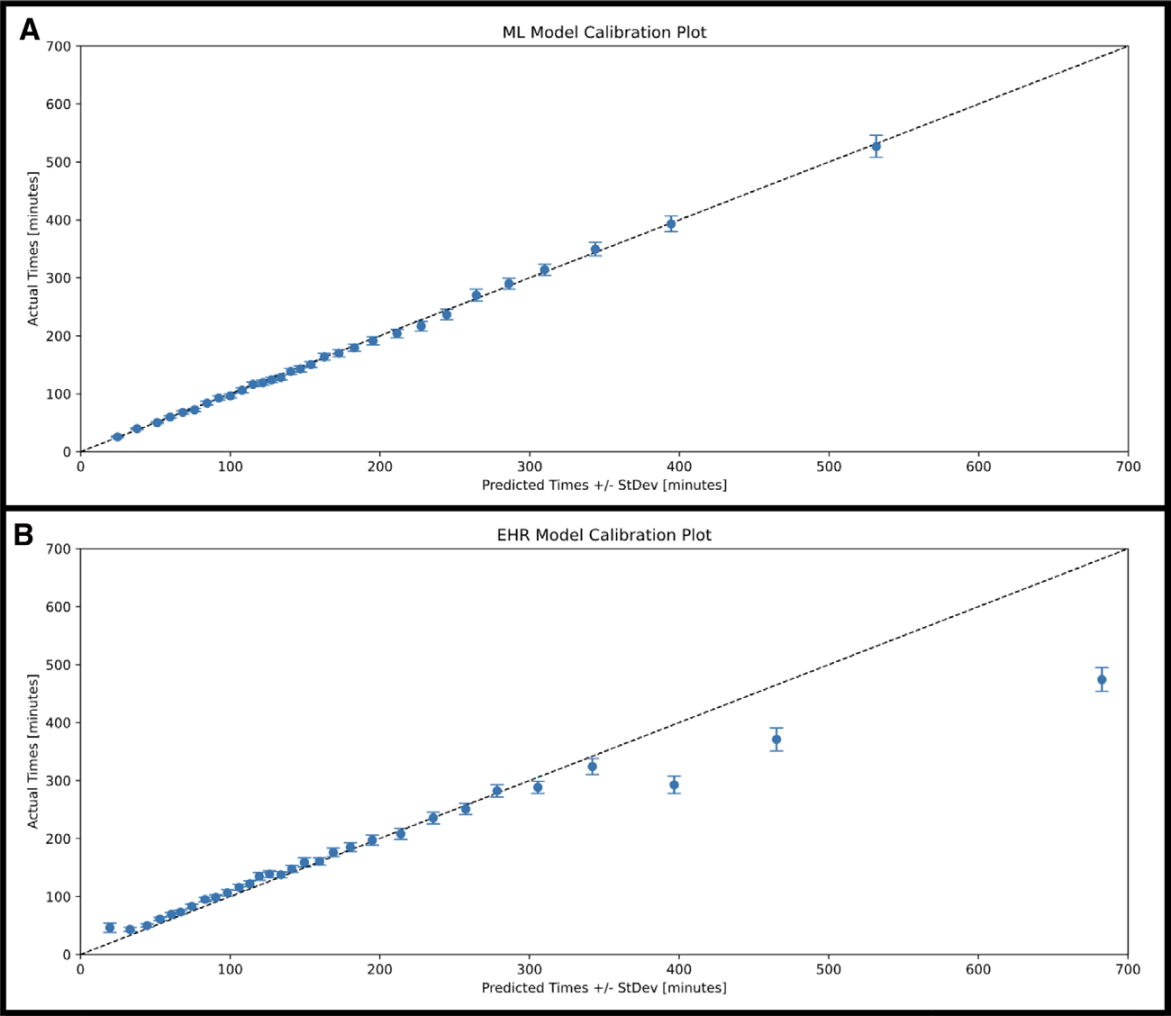
Calibration plots using quantile binning (*q* = 30) comparing predicted and actual operative times for the ML model (A) and EHR-based estimates (B). Error bars indicate the standard deviation of the predicted times within each quantile bin. Both models demonstrate slight over-prediction for shorter cases and underestimation for longer cases. The EHR model shows much more narrow error bars across all bins, indicating more stable estimates than the ML model.

We developed 3 models to predict the 3 nonoverlapping phases of the surgery – preprocedure, operative, and postprocedure. Hyperparameter tuning aided in the selection of the best model (Supplemental Tables 2–4, https://links.lww.com/AOSO/A576). The models predicting preprocedure and postprocedure times showed less RMSE and MAE compared to the operative model, while the preprocedure and operative models had higher *R*^2^ value compared to the postprocedure model (Supplemental Figure 3, https://links.lww.com/AOSO/A576). We then tested our models using a temporal holdout set, by aggregating all 3 model predictions to estimate in-room to out-room time and then compared to the embedded EHR model. The ML model significantly outperformed the EHR model with an RSME of 61.0 versus 91.0 minutes (*P* < 0.01), MAE of 39.6 versus 51.8 minutes (*P* < 0.01) and *R*^2^ of 0.78 versus 0.50 (*P* < 0.01), respectively (Fig. 1). Calibration plots show the relationship between predicted and observed case lengths for the ML model (Fig. 2A) and the EHR (Fig. 2B). Case length prediction accuracy of the ML model outperformed the EHR model, especially for shorter cases and those that are very long (Fig. 2). Model performance was stable in both the elective and emergent subgroups (Supplemental Figure 4, https://links.lww.com/AOSO/A576).

### Feature Importance

Across all 3 models, historical case durations were the highest-scoring features within their respective estimation contexts (eg, preoperative historical times were most important for the preoperative model). These times represent the data available at least 2 days before surgery. Surgeon RVUs in the last 90 days, Surgeon RVU volume ratio, and patient-level factors (age, BMI, claims fragility index, and social determinates of health) also consistently contributed to each of the models’ performance (Supplemental Figure 5, https://links.lww.com/AOSO/A576).

### Model Impact

**FIGURE 3.**
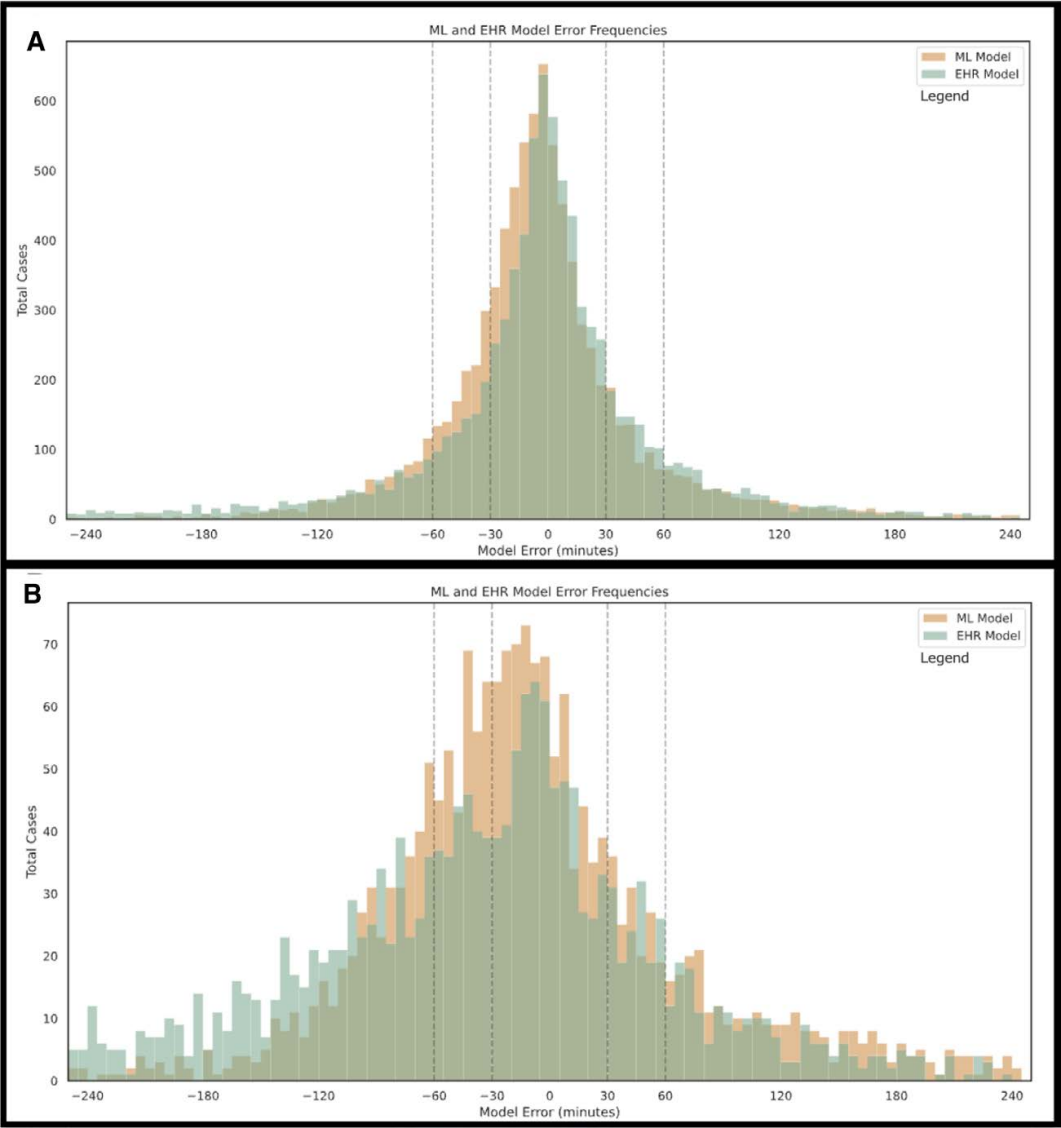
Overlaid histograms of ML model versus EHR performance in (A) all cases and in (B) the subgroup without historical data.

When compared to the actual case duration, our model predicted 213 more cases (4% increase) within ±30 minutes of the actual duration compared to the EHR model, corresponding to 66 additional OR hours falling within this 4-month period. The most notable improvement was observed in reducing underestimation error. For cases underestimated by more than 60 minutes, our model reduced the total number of underestimated OR hours by 1832 and decreased the proportion of severely underestimated cases from 13.8% (1195 cases) to 9.7% (841 cases) when compared to the EHR model (Fig. 3).

**FIGURE 4.**
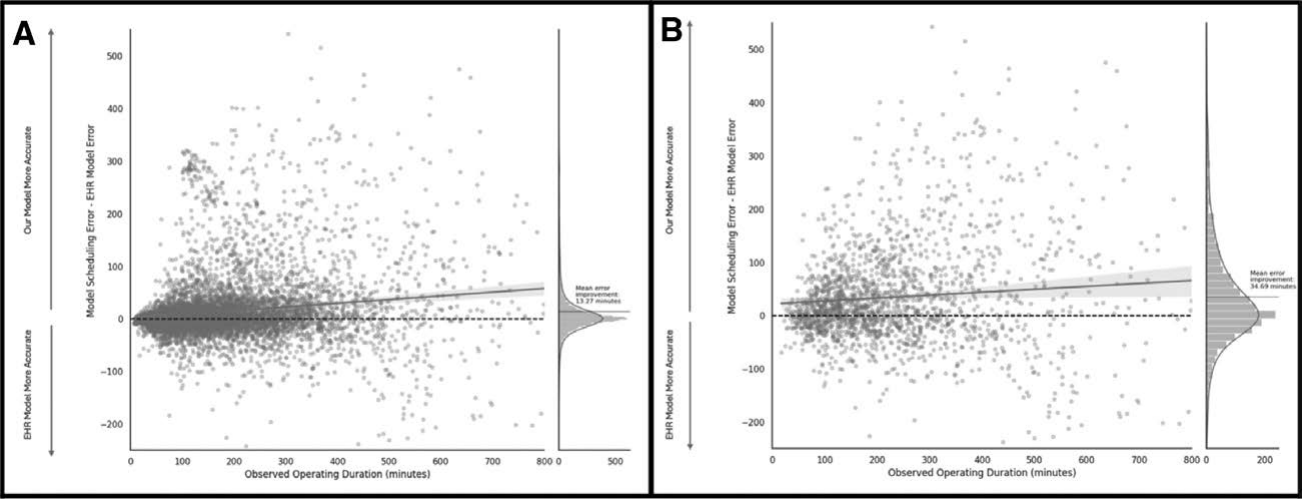
Improvement in scheduling accuracy by the ML model compared to the EHR, stratified by observed operating duration. (A) All surgical cases. (B) Subset of case types without historical cases. Scatter plots show the difference in absolute scheduling error between our model and the EHR (*y*-axis) as a function of actual case duration (*x*-axis). Positive values indicate that the ML model was more accurate than the EHR; negative values indicate better EHR model performance. Each point represents a surgical case. A linear trend line with 95% confidence interval (shaded band) summarizes the relationship between case duration and model improvement. Marginal histograms display the distribution of case-level differences, with mean error improvement values noted. In both datasets, the ML model demonstrates a net improvement over the EHR, with larger gains for longer cases and those without historical data. (A) Slope: 1.3, *y*-intercept: 0.07; (B) Slope: 21.7, *y*-intercept: 0.05. Positive slopes in both graphs are statistically significant (*P* < 0.01).

When evaluating predictive performance for the case subgroup without historical data, the ML model demonstrated a greater relative improvement over the EHR model than when historical data was included. In cases without historical data, the ML model accurately predicted 35% of cases of cases within ±30 minutes versus 29% for the EHR model (*P* < 0.01). For cases with historical data, the ML model provides marginal improvement, correctly predicting 59% of cases within ±30 minutes, compared to 56% for the EHR model (*P* < 0.01). A similar trend was observed for underestimations greater than 60 minutes, where the ML model outperformed the EHR model by a larger margin when historical data was removed. With historical data, the ML model underestimated fewer cases (10% vs 14%, *P* < 0.01), and when historical data was excluded, its advantage increased (23% vs 37%, *P* < 0.01) (Fig. 3). Notably, the ML model resulted in a mean error improvement of 13 minutes compared to the EHR model. This improvement was particularly evident in cases without historical data, where the mean error reduction was 35 minutes (Fig. 4). Therefore, across the 4-month holdout period, the ML model reduces case scheduling error by 1908 hours and 1110 hours compared to the EHR model for all cases and the subgroup of cases without historical time estimates, respectively.

## DISCUSSION

This study demonstrates the development and utility of a machine learning model for predicting operative case length across multiple surgical specialties at a quaternary care hospital within a complex health system. The ML model significantly outperforms the embedded EHR model in predicting surgical case duration. These findings suggest that the implementation of a ML model can improve case length estimation, which holds high potential value for perioperative and surgical teams.

Traditional case scheduling methods reliant on simplistic historical averages or heuristic-based estimates can lead to inaccuracies that compromise OR efficiency, negatively affecting operation costs and patient and provider satisfaction.^22–24^ ML techniques offer a solution to these limitations by systematically integrating multidimensional data and identifying nonlinear relationships, otherwise missing from traditional analysis, thus justifying the adoption of ML for surgical case duration prediction. The ML model presented here incorporates expansive information pertaining to patient demographics, comorbidities, socioeconomic factors, provider-specific metrics, and operational characteristics, offering improved predictive capabilities against simpler approaches. ML model performance is especially important for shorter case length estimation, which is often of greatest value for ambulatory settings.

Previous research has explored the utility of ML models for predicting surgical durations with varying success, often limited by their narrow scope—either confined to a single specialty^12^ or restricted to limited data points.^25^ For instance, 1 study effectively utilized ML algorithms, incorporating unstructured text and preoperative clinical variables to predict operative duration in pediatric surgical cases, significantly outperforming traditional scheduling methods. However, this study used features that are not immediately extractable the day before surgery at our institution (eg, anesthesia severity assessment rating) and included features that require manual input (eg, scheduled duration) In contrast, our approach covers a wide range of complex surgical services without manual adjustment of case estimates and uses features only known 1 or more days before surgery. Therefore, our approach can be directly compared to the embedded EHR model, which faces similar constraints.

Analysis of feature importance revealed that historical case times, surgeon experience, and number of panels were among the most important features in all 3 models. This is consistent with previously published machine learning models that identified previous case-time duration, procedure ID, and surgeon characteristics as important features for accurate case-length estimation.^4,11,26^ Our model’s predictive performance additionally relied on patient-level clinical variables such as patient age, multimorbidity indices, BMI, and claims-based fragility index. These factors align with known determinants of surgical complexity and variability.^27,28^

Our model outperformed the EHR model in case length estimation, leading to a reduction in total error and fewer cases misestimated by more than ±30 minutes and ±60 minutes. Our model most notably reduced the number of underestimations compared to the EHR model. Underestimation leads to OR overutilization, which causes increases in staff overtime and prolonged patient wait times.^29,30^ Further, the EHR model relies heavily on historical case averages, which are useful for common and straightforward cases but are insufficient for new or rare cases to the hospital, highlighting the need for ML to infer times from other factors^24^ Indeed, the ML model significantly outperformed the EHR model when estimating case lengths without historical data, demonstrating greater versatility and applicability for these challenging cases.

We demonstrated significant improvement in our case length modeling program across multiple specialties and operating facilities in the health system. Future directions include the deployment of this model to perioperative teams throughout other hospitals in our health system to improve OR scheduling and efficiency.^31^ Further, deep learning advancements have enabled higher-order data capture and analytic techniques of surgical workflows, allowing models to integrate to minute-to-minute intraoperative data.^32^ This integration allows real-time prediction updates, a feature that could be incorporated into future iterations of model development.

This model was developed to be seamlessly integrated into the existing perioperative workflow, providing decision support for our OR scheduling teams. The ML model is currently deployed and predictions surface within the EHR interface, allowing staff to prospectively adjust case times. A subsequent study will be designed to rigorously evaluate the real-world impact of this intervention on key performance indicators, including patient wait times, OR throughput, on-time starts, and overall efficiency, to fully assess its value in a clinical setting.

### Limitations

Several limitations must be acknowledged. First, this study was conducted at a single quaternary care center, which may limit generalizability to other institutions with different patient demographics and surgical practices. Differences in OR workflows, staffing, and technology adoption may affect model performance in different environments. However, across our health system, the models met or exceed current scheduling accuracy at 4 out of our 5 hospitals, suggesting that the model can scale and be disseminated across different hospital settings. Second, although the ML model demonstrated strong predictive performance, it is dependent on the quality of data available in the EHR. Although we employed significant data cleaning to remove “illogical” timestamps, other missing or inaccurate documentation could impact model reliability. Third, this model is intended for perioperative services, not other teams. For our model, general OR staff, including nursing, residents, and anesthesia, are not staffed until the night before, so we could not use personnel information. Fourth, details about the actual operation cannot be included in the model because they are often unknown at the time of scheduling. Finally, the model is retrained at regular intervals to avoid drift. It is infeasible to include small changes in the modeling across time in a manuscript; however, it is critical that all ML models applied in the healthcare setting be assessed for drift over time and updated to ensure reliability.

## CONCLUSION

A robust machine learning-based predictive model for surgical case length significantly improves perioperative planning. Healthcare systems can optimize scheduling, enhance efficiency, and improve patient care by utilizing advanced analytics. Future studies should refine models to maximize clinical utility and applicability, incorporating real-time adjustments and crossinstitutional validation. This study supports machine learning perioperative management, aiming to improve surgical care delivery, reduce costs, and enhance experiences.

## ACKNOWLEDGMENTS

J.W.R.: Manuscript drafting and editing, data visualization, and data analysis. I.J.P.: Model development, data visualization and validation, and data analysis. D.W.G.: Data interpretation. A.A.N.: Data interpretation. C.T.B.: Data curation and visualization. C.C.H.: Data acquisition and model development. K.P.: Project design. B.K.: Data acquisition. J.H.K.: Project conception and design. G.E.W.: Project conception and design, data analysis, and interpretation. R.R.K.: Project conception and design, data analysis, and interpretation. All authors made major contributions in revising the drafted manuscript. All authors approve publication of this manuscript version as stated in the cover letter.

## Supplementary Material
