# Supplementary material for: Development and Evaluation of an Operative Case Length Prediction Model in Adult Surgical Patients
Source: Ann Surg Open. 2026 Feb 9;7(1):e652. doi: 10.1097/AS9.0000000000000652 (PMC13016189; doi:10.1097/AS9.0000000000000652)
Supplement: Supplementary file 1 [file as9-7-e652-s001.pdf]

Supplemental Digital Content 1:

for

Development and Evaluation of an Operative Case Length Prediction Model in Adult Surgical Patients

J. Walker Rosenthal, BS
Isaac J. Perron, PhD
Drew W. Goldberg, MD
Armaan A. Nallicheri, BA
Charles T. Bradford, MS
Charles C. Horn, PhD
Kaley Piersanti, BA
Bhavana Kunisetty, BA
John H. Keogh, MD
Gary E. Weissman, MD
Rachel R. Kelz, MD

Table of Contents

Supplementary Methods………………………………………………………………………………. pg. 3

Supplementary Figure 1………………………………………………………………………………. pg. 4

Supplementary Figure 2………………………………………………………………………………. pg. 5

Supplementary Figure 3………………………………………………………………………………. pg. 6

Supplementary Figure 4………………………………………………………………………………. pg. 7

Supplementary Table 1……………………………………………………………………………….. pg. 8

Supplementary Table 2……………………………………………………………………………….. pg. 13

Supplementary Table 3……………………………………………………………………………….. pg. 17

Supplementary Table 4……………………………………………………………………………….. pg. 21

Supplementary Table 5……………………………………………………………………………….. pg. 25

Supplementary Table 6……………………………………………………………………………….... pg. 27

Supplemental Methods

Definition of Illogical Timestamps

Illogical timestamps were defined in collaboration with perioperative services by first identifying key timestamps for each surgery: 1) preoperative care completed; 2) patient in room; 3) anesthesia start; 4) procedure start; 5) procedure end; 6) anesthesia end; 7) patient out room; 8) post-anesthesia recovery completed. Next, timestamp comparisons ensured these discrete events happened in sequence, with additional criteria: A) patient in room could occur up to 15 minutes before preoperative care completed; B) anesthesia start must occur between 15 minutes before and 180 minutes after patient in room; C) procedure start must occur within 720 minutes after patient in room; D) anesthesia end must occur either before patient out room or within 30 minutes after; and E) patient out room must occur within 720 minutes after procedure end. The only required timestamps were patient in room (2), procedure start (4), procedure end (5), and patient out room (7). If any of the non-essential timestamps were missing, the above logic was ignored. The presence of grossly inaccurate timestamps can affect model training and therefore must be removed from the dataset.

Hyperparameter tuning and Cross Validation

A full grid search across the following hyperparameters were tested: max depth (-1,5,30); number of leaves (30,150,300); minimum child samples (20,50); number of estimators (100,200); minimum gain per split (0.001, 0.01). Results from the full grid are reported in Supplementary Table S4. We used 5-times repeated, 10-fold cross validation to establish reliable performance estimates for each model tested. With this approach, the training data is randomly shuffled into 10 buckets, stratified by surgical service. Then each bucket is sequentially held out ("CV test") while the remaining 90% of data is used to fit the model ("CV train"), thus generating 10 models with measurable performance predicting CV train and CV test. We repeat this process 5 times, for a total of 50 model fits for each hyperparameter set. The entire training set was used for cross-validation. We include model performance for both the CV train and CV test as an indication of over-fitting. We chose the best hyperparameters by considering the following factors: 1) lowest statistically equivalent RMSE on CV test data; 2) similar RMSE between CV train vs CV test to reduce overfitting; and 3) least complex hyperparameters for simplest model (e.g., fewer trees and nodes, less depth).

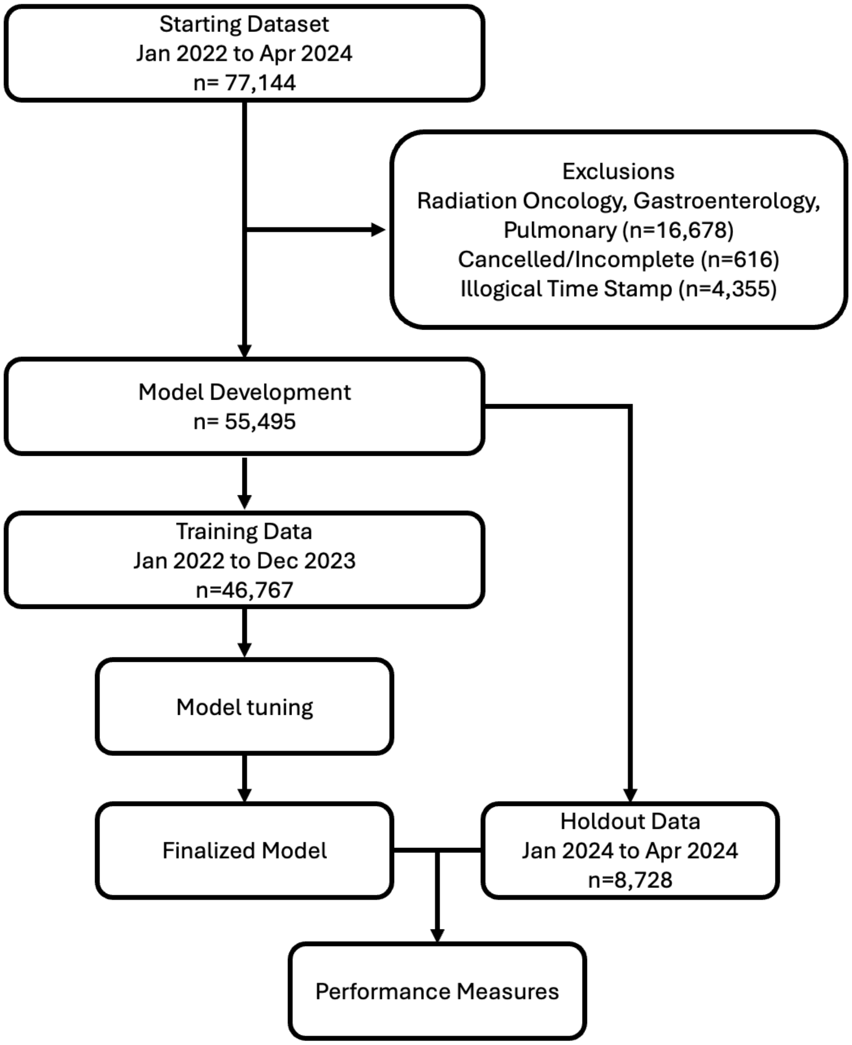

Supplementary Figure 1. Consort Diagram

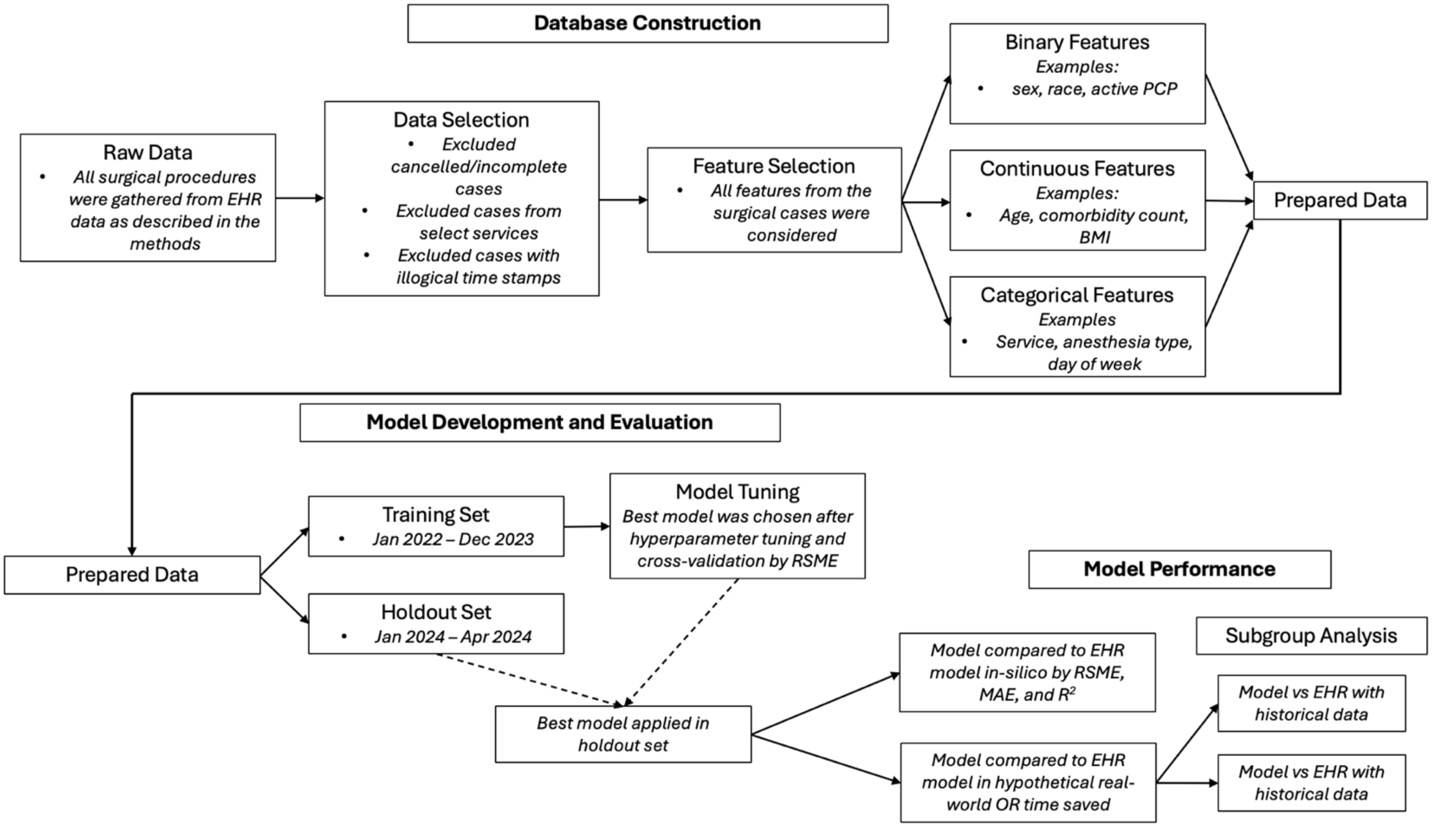

Supplementary Figure 2. Overview of database construction, model development, and performance evaluation pipeline.

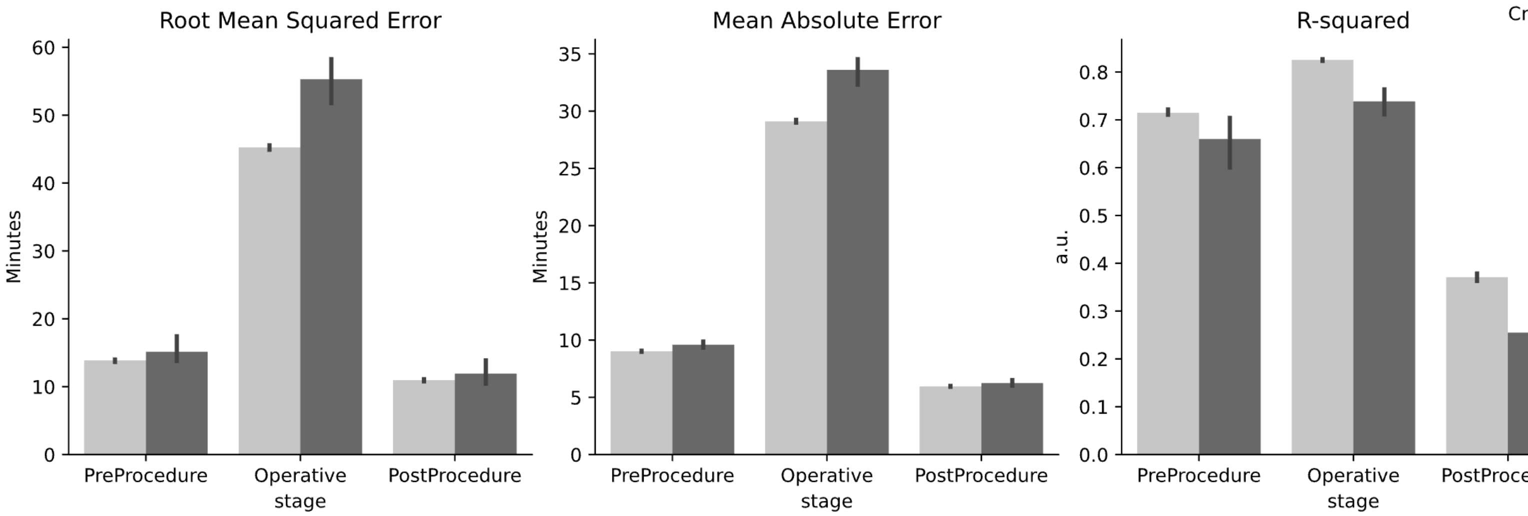

Supplementary Figure 3. Model validation and performance compared to EHR in-silico.
(A) Cross-validation performance of our model by perioperative stage. Models were evaluated on training and test folds using root mean squared error, mean absolute error, and R-squared. The operative stage had the largest prediction error, but also the highest R^2. Performance was consistent between training and test folds. Error bars represent ± 1 standard deviation.

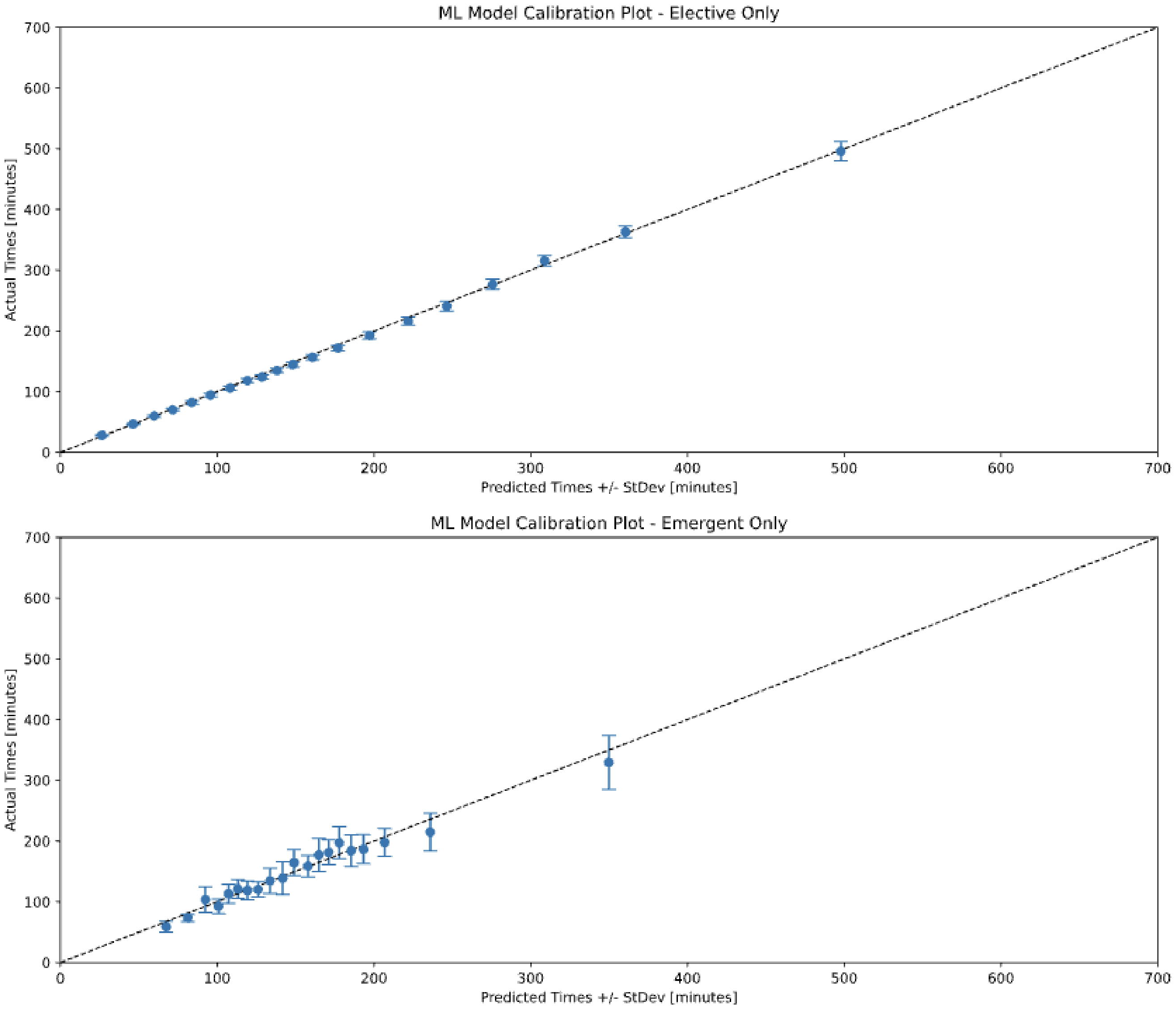

Supplementary Figure 4. Calibration plot of ML Model in the elective and emergent subgroups.

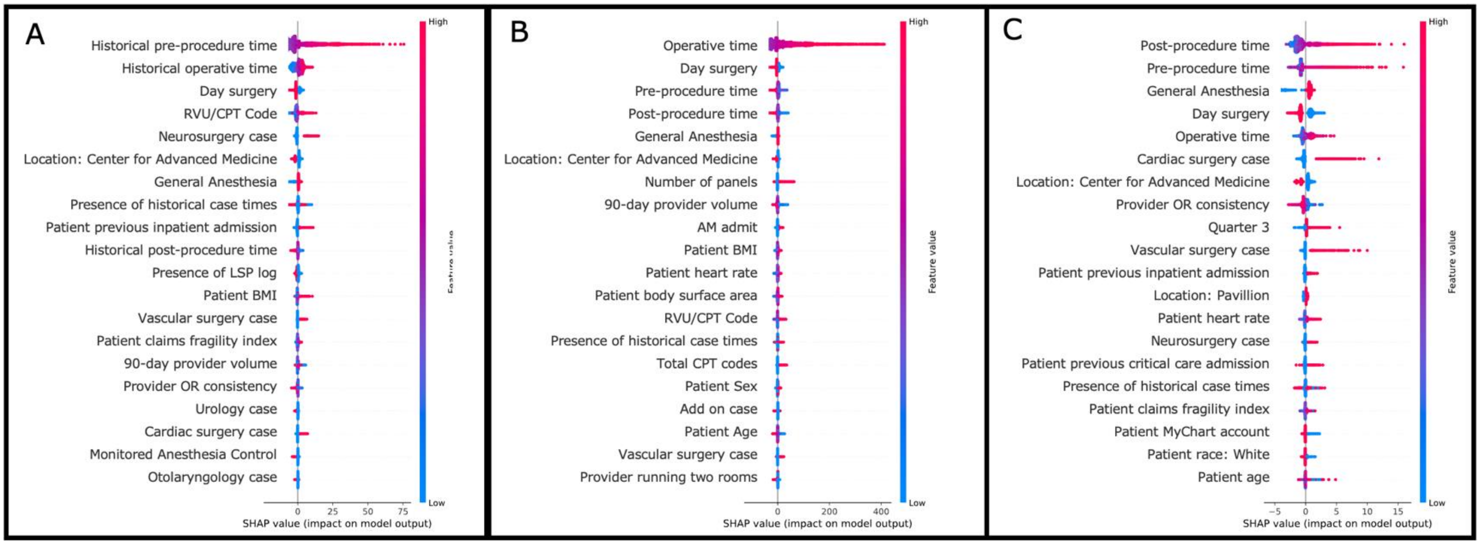

Supplementary Figure 5. Feature importance for using Shapley plots.

Shapley plots to estimate the importance of individual features in each model: (A) Pre-procedure time, (B) Operative time, and (C) Post-procedure time. Red color indicates higher feature values and blue color indicates lower feature values, e.g., for binary features, 1 is red and 0 is blue. High SHAP values indicate that the features influence the model to predict a longer case, while low SHAP values decrease the model estimate output. For example, historical case averages for pre-procedure time, operative time, and post-procedure time were the most important features for each model respectively, with a positive association between the feature and the outcome.

Supplementary Table 1: Model features and descriptions

Variable Name	Type	Description
Add-On Case	Binary	Indicates whether the surgery was an add-on case.
Managed Care	Binary	Primary insurance coverage through a managed care plan.
Medicare	Binary	Primary insurance coverage through Medicare.
Medicaid	Binary	Primary insurance coverage through Medicaid.
Self-Pay	Binary	Patient is self-paying for the encounter.
Sex	Binary	Patient sex (Male or Female).
Race: White	Binary	Patient self-identified race as White.
Race: Black	Binary	Patient self-identified race as Black.
Race: Asian	Binary	Patient self-identified race as Asian.
Race: Hispanic	Binary	Patient self-identified ethnicity as Hispanic.
Race: Multiple	Binary	Patient self-identified as more than one race.
English Speaking	Binary	Patient reported English as their primary language.
Marital Status: Married	Binary	Patient is currently married.
Active PCP	Binary	Patient has an active primary care provider.
Active MyChart	Binary	Patient has an active MyChart account.
Health Literacy Risk	Binary	Patient flagged as having potential health literacy challenges.
Housing Risk	Binary	Patient flagged as having unstable housing.
Social Isolation Risk	Binary	Patient flagged as being at risk for social isolation.
Succinylcholine Allergy	Binary	Patient has a documented succinylcholine allergy.
Latex Allergy	Binary	Patient has a documented latex allergy.
Multimorbidity	Binary	Patient has multiple chronic conditions.
Anesthesia Complexity	Binary	Patient has conditions that increase anesthesia complexity.
Running Two Rooms	Binary	Surgeon had overlapping cases in two operating rooms.
Previous Emergency Encounter	Binary	Patient had a previous emergency department encounter, prior 90 days
Previous Inpatient Encounter	Binary	Patient had a previous inpatient admission, prior 90 days
AIDS	Binary	Elixhauser comorbidity index flag for aids.
Alcohol Use	Binary	Elixhauser comorbidity index flag for alcohol use.
Iron Deficiency Anemia	Binary	Elixhauser comorbidity index flag for iron deficiency anemia.
Autoimmune Disease	Binary	Elixhauser comorbidity index flag for autoimmune disease.
Blood Loss Anemia	Binary	Elixhauser comorbidity index flag for blood loss anemia.
Leukemia	Binary	Elixhauser comorbidity index flag for leukemia.
Lymphoma	Binary	Elixhauser comorbidity index flag for lymphoma.
Metastatic Cancer	Binary	Elixhauser comorbidity index flag for metastatic cancer.
Cancer In Situ	Binary	Elixhauser comorbidity index flag for cancer in situ.
Solid Tumor Cancer	Binary	Elixhauser comorbidity index flag for solid tumor cancer.

Cerebrovascular Disease	Binary	Elixhauser comorbidity index flag for cerebrovascular disease.
Coagulopathy	Binary	Elixhauser comorbidity index flag for coagulopathy.
Dementia	Binary	Elixhauser comorbidity index flag for dementia.
Depression	Binary	Elixhauser comorbidity index flag for depression.
Diabetes with Complications	Binary	Elixhauser comorbidity index flag for diabetes with complications.
Diabetes without Complications	Binary	Elixhauser comorbidity index flag for diabetes without complications.
Drug Abuse	Binary	Elixhauser comorbidity index flag for drug abuse.
Heart Failure	Binary	Elixhauser comorbidity index flag for heart failure.
Hypertension with Complications	Binary	Elixhauser comorbidity index flag for hypertension with complications.
Hypertension without Complications	Binary	Elixhauser comorbidity index flag for hypertension without complications.
Mild Liver Disease	Binary	Elixhauser comorbidity index flag for mild liver disease.
Severe Liver Disease	Binary	Elixhauser comorbidity index flag for severe liver disease.
Chronic Lung Disease	Binary	Elixhauser comorbidity index flag for chronic lung disease.
Movement Disorder	Binary	Elixhauser comorbidity index flag for movement disorder.
Other Neurological Disorder	Binary	Elixhauser comorbidity index flag for other neurological disorder.
Seizure Disorder	Binary	Elixhauser comorbidity index flag for seizure disorder.
Obesity	Binary	Elixhauser comorbidity index flag for obesity.
Paralysis	Binary	Elixhauser comorbidity index flag for paralysis.
Peripheral Vascular Disease	Binary	Elixhauser comorbidity index flag for peripheral vascular disease.
Psychoses	Binary	Elixhauser comorbidity index flag for psychoses.
Pulmonary Circulation Disorder	Binary	Elixhauser comorbidity index flag for pulmonary circulation disorder.
Moderate Renal Failure	Binary	Elixhauser comorbidity index flag for moderate renal failure.
Severe Renal Failure	Binary	Elixhauser comorbidity index flag for severe renal failure.
Hypothyroidism	Binary	Elixhauser comorbidity index flag for hypothyroidism.
Other Thyroid Disorder	Binary	Elixhauser comorbidity index flag for other thyroid disorder.
Peptic Ulcer Disease	Binary	Elixhauser comorbidity index flag for peptic ulcer disease.
Valvular Disease	Binary	Elixhauser comorbidity index flag for valvular disease.
Weight Loss	Binary	Elixhauser comorbidity index flag for weight loss.
BMI (Most Recent)	Continuous	Patient’s most recently recorded body mass index.

CFI Weight	Continuous	Weighted value from the clinical frailty index.
Comorbidity Count	Continuous	Total number of comorbidities.
Driving Distance (miles)	Continuous	Distance from patient residence to hospital.
Total Elixhauser Score	Continuous	Total score based on Elixhauser comorbidity index.
Historical Pre-Procedure Time Average	Continuous	Historical time averages from patient entering the OR to start of surgery.
Historical Operative Time Average	Continuous	Historical time averages from surgeon "cut" to "close".
Historical Post-Procedure Time Average	Continuous	Historical time averages from end of surgery to patient out of OR.
MAP (Most Recent)	Continuous	Most recent mean arterial pressure.
Number of Panels	Continuous	Number of panels involved in the case.
Total Allergies	Continuous	Count of patient-reported allergies.
Previous Critical Care Encounters	Continuous	Number of prior critical care visits, prior 90 days.
Previous Inpatient Admissions	Continuous	Number of prior inpatient admissions, prior 90 days
Provider Volume (90 Days)	Continuous	Number of procedures performed by provider in prior 90 days measured by RVUs
Provider OR ID Consistency	Continuous	Number of OR procedures the primary surgeon completed in the specified OR, past 365 days.
Pulse (Most Recent)	Continuous	Most recent recorded pulse rate.
Respiratory Rate (Most Recent)	Continuous	Most recent recorded respiratory rate.
SDoH: Below Poverty Line (%)	Continuous	Percent of residents below the federal poverty line.
SDoH: Gini Index	Continuous	Income inequality index for patient's area.
SDoH: Median Income by Race	Continuous	Median income in patient's area stratified by race.
SDoH: Median Rent	Continuous	Median gross rent in the patient's census tract.
SDoH: Medicaid Coverage (%)	Continuous	Percent of residents with Medicaid in patient's area.
SDoH: No Vehicle Access (%)	Continuous	Percent of households without access to a vehicle.
SDoH: Limited English Proficiency (%)	Continuous	Percentage of residents with limited English in patient's area.
SDoH: Unemployment Rate (%)	Continuous	Unemployment rate in the patient's residential area.
SDoH: Uninsured Rate (%)	Continuous	Percent of residents without health insurance.
SDoH: Vacant Housing (%)	Continuous	Percentage of housing units that are vacant.
Q1	Categorical	Indicates that the encounter occurred in the first quarter (Jan-Mar).
Q2	Categorical	Indicates that the encounter occurred in the second quarter (Apr-Jun).
Q3	Categorical	Indicates that the encounter occurred in the third quarter (Jul-Sep).
Q4	Categorical	Indicates that the encounter occurred in the fourth quarter (Oct-Dec).
Mon	Categorical	Surgery scheduled on Monday.
Tue	Categorical	Surgery scheduled on Tuesday.

Wed	Categorical	Surgery scheduled on Wednesday.
Thu	Categorical	Surgery scheduled on Thursday.
Fri	Categorical	Surgery scheduled on Friday.
COLD	Categorical	Cold case scheduling.
ELECTIVE	Categorical	Elective surgery.
HOT	Categorical	Hot case scheduling.
NOW	Categorical	Immediate/emergent scheduling.
POSTPONEDC	Categorical	Postponed case.
URGENT	Categorical	Urgent surgery designation.
BRS	Categorical	Breast Surgery
CARDSURG	Categorical	Cardiac Surgery
CARDVASC	Categorical	Cardiovascular Surgery
CRS	Categorical	Colorectal Surgery
EOS	Categorical	Endocrine & Oncologic Surgery
GIS	Categorical	Gastrointestinal Surgery
GYN	Categorical	Gynecology
NEUROSURG	Categorical	Neurosurgery
OMFS	Categorical	Oral and Maxillofacial Surgery
ORL	Categorical	Otolaryngology (ENT)
ORLHN	Categorical	Otolaryngology - Head and Neck Surgery
ORTHO	Categorical	Orthopedic Surgery
PLASSURG	Categorical	Plastic Surgery
THORSURG	Categorical	Thoracic Surgery
TRANSPLANT	Categorical	Transplant Surgery
TRAUMA	Categorical	Trauma Surgery
UROLOGY	Categorical	Urologic Surgery
VASCSURG	Categorical	Vascular Surgery
OTHER	Categorical	All Other services
Operating Room ID	Categorical	Operating room assigned to the procedure.
Hospital ID	Categorical	Surgical facility where the procedure occurred.
Primary Physician ID	Categorical	Identifier for the attending or primary physician.
BLO	Categorical	Block anesthesia used.
EPI	Categorical	Epidural anesthesia used.
GEN	Categorical	General anesthesia used.

IVCS	Categorical	Intravenous conscious sedation.
LOC	Categorical	Local anesthesia.
MAC	Categorical	Monitored anesthesia care.
None	Categorical	No anesthesia recorded.
SPI	Categorical	Spinal anesthesia.
AM Admit	Categorical	Morning admission.
Day Surgery	Categorical	Admitted for day surgery.
Direct Admit	Categorical	Direct admission (not through ED).
Emergency	Categorical	Emergency department admission.
Endoscopic Bronchoscopy	Categorical	Endoscopy/Bronchoscopy admission.
Inpatient	Categorical	Standard inpatient admission.

Supplementary Table 2: Pre-procedure model hyperparameter tuning results.

Mean fit time (SD)	Mean score time (SD)	Mean test score (SD)	Mean train score (SD)	max depth	Leaves (n)	minimum child samples	learning rate	Estimators (n)	minimum split gain
2.3218 (0.1191)	0.084 (0.0034)	-15.1789 (0.9617)	-13.4934 (0.0761)	-1	30	20	0.1	100	0.001
4.0077 (0.3489)	0.0889 (0.0038)	-15.1932 (0.9471)	-10.0673 (0.0614)	-1	150	20	0.1	100	0.001
5.9466 (0.3409)	0.094 (0.0057)	-15.2899 (0.9611)	-8.0505 (0.0852)	-1	300	20	0.1	100	0.001
2.8717 (0.1541)	0.0888 (0.0044)	-15.1175 (0.9657)	-12.5021 (0.0749)	-1	30	20	0.1	200	0.001
6.0478 (0.5784)	0.0984 (0.0042)	-15.2726 (0.9504)	-8.0042 (0.0656)	-1	150	20	0.1	200	0.001
9.2879 (0.3223)	0.1102 (0.0045)	-15.4131 (0.9697)	-5.577 (0.0803)	-1	300	20	0.1	200	0.001
2.3772 (0.1333)	0.0845 (0.0044)	-15.1785 (0.9575)	-13.4933 (0.0801)	-1	30	20	0.1	100	0.01
3.9238 (0.1906)	0.0882 (0.0047)	-15.1976 (0.9618)	-10.067 (0.0626)	-1	150	20	0.1	100	0.01
5.7583 (0.3169)	0.0921 (0.0037)	-15.2921 (0.964)	-8.053 (0.0869)	-1	300	20	0.1	100	0.01
2.8889 (0.1658)	0.0888 (0.0048)	-15.1227 (0.9577)	-12.4999 (0.0786)	-1	30	20	0.1	200	0.01
5.778 (0.2527)	0.0983 (0.0049)	-15.2711 (0.9665)	-8.02 (0.0618)	-1	150	20	0.1	200	0.01
9.2346 (0.3586)	0.1107 (0.0051)	-15.4218 (0.9676)	-5.5868 (0.0801)	-1	300	20	0.1	200	0.01
2.1785 (0.188)	0.0842 (0.004)	-15.1498 (0.9541)	-13.7645 (0.0874)	-1	30	50	0.1	100	0.001

3.3762 (0.1713)	0.0738 (0.004)	-15.1684 (0.9513)	-10.7954 (0.0947)	-1	150	50	0.1	100	0.001
4.7613 (0.2691)	0.079 (0.0036)	-15.2371 (0.9506)	-9.1715 (0.1163)	-1	300	50	0.1	100	0.001
2.6032 (0.101)	0.0727 (0.0034)	-15.1023 (0.9545)	-12.8481 (0.0817)	-1	30	50	0.1	200	0.001
4.9812 (0.1861)	0.0847 (0.0047)	-15.264 (0.9564)	-8.7698 (0.0887)	-1	150	50	0.1	200	0.001
7.7881 (0.3178)	0.1076 (0.0057)	-15.3891 (0.9454)	-6.7028 (0.1361)	-1	300	50	0.1	200	0.001
2.1517 (0.1043)	0.0684 (0.0037)	-15.1492 (0.9513)	-13.7661 (0.086)	-1	30	50	0.1	100	0.01
3.1415 (0.115)	0.0728 (0.0035)	-15.1664 (0.9618)	-10.7986 (0.0989)	-1	150	50	0.1	100	0.01
5.1466 (4.0256)	0.0788 (0.0042)	-15.245 (0.9466)	-9.1831 (0.1199)	-1	300	50	0.1	100	0.01
2.7009 (0.1162)	0.0737 (0.004)	-15.1002 (0.9508)	-12.846 (0.0776)	-1	30	50	0.1	200	0.01
5.3313 (0.1721)	0.0853 (0.0039)	-15.2679 (0.9649)	-8.7827 (0.0981)	-1	150	50	0.1	200	0.01
8.2092 (0.3259)	0.1101 (0.0059)	-15.3942 (0.9427)	-6.7166 (0.1343)	-1	300	50	0.1	200	0.01
2.0902 (0.0718)	0.0663 (0.0033)	-15.2706 (0.9456)	-14.399 (0.1013)	5	30	20	0.1	100	0.001
2.1003 (0.082)	0.0668 (0.0042)	-15.2731 (0.9503)	-14.3946 (0.102)	5	150	20	0.1	100	0.001
2.0901 (0.0633)	0.0667 (0.0032)	-15.2731 (0.9503)	-14.3946 (0.102)	5	300	20	0.1	100	0.001
2.4165 (0.1279)	0.0694 (0.005)	-15.1588 (0.9497)	-13.876 (0.1007)	5	30	20	0.1	200	0.001
2.4079 (0.0993)	0.0691 (0.0033)	-15.1576 (0.959)	-13.8706 (0.1021)	5	150	20	0.1	200	0.001

2.4131 (0.1433)	0.0689 (0.0029)	-15.1576 (0.959)	-13.8706 (0.1021)	5	300	20	0.1	200	0.001
2.0741 (0.0617)	0.0658 (0.003)	-15.2712 (0.9467)	-14.4006 (0.1002)	5	30	20	0.1	100	0.01
2.0718 (0.0733)	0.0663 (0.0038)	-15.2704 (0.9503)	-14.3944 (0.1018)	5	150	20	0.1	100	0.01
2.0759 (0.0519)	0.0667 (0.0058)	-15.2704 (0.9503)	-14.3944 (0.1018)	5	300	20	0.1	100	0.01
2.4401 (0.0972)	0.0702 (0.0034)	-15.155 (0.9517)	-13.879 (0.1021)	5	30	20	0.1	200	0.01
2.3961 (0.0674)	0.0695 (0.004)	-15.1552 (0.9569)	-13.8702 (0.1003)	5	150	20	0.1	200	0.01
2.4297 (0.1306)	0.0696 (0.0035)	-15.1552 (0.9569)	-13.8702 (0.1003)	5	300	20	0.1	200	0.01
1.9948 (0.0714)	0.0665 (0.0036)	-15.2503 (0.943)	-14.5847 (0.1035)	5	30	50	0.1	100	0.001
1.9794 (0.1227)	0.0655 (0.004)	-15.248 (0.945)	-14.5817 (0.1037)	5	150	50	0.1	100	0.001
1.9965 (0.0709)	0.0671 (0.0041)	-15.248 (0.945)	-14.5817 (0.1037)	5	300	50	0.1	100	0.001
2.3853 (0.2026)	0.0713 (0.0044)	-15.129 (0.9526)	-14.1466 (0.1009)	5	30	50	0.1	200	0.001
2.3423 (0.1411)	0.0711 (0.0048)	-15.1312 (0.9509)	-14.1384 (0.1047)	5	150	50	0.1	200	0.001
2.2868 (0.1084)	0.0698 (0.0036)	-15.1312 (0.9509)	-14.1384 (0.1047)	5	300	50	0.1	200	0.001
1.9957 (0.0686)	0.0668 (0.0035)	-15.2514 (0.9407)	-14.5856 (0.1052)	5	30	50	0.1	100	0.01
2.0025 (0.1193)	0.0672 (0.0033)	-15.2459 (0.9455)	-14.5817 (0.1028)	5	150	50	0.1	100	0.01

1.9837 (0.0663)	0.0667 (0.0039)	-15.2459 (0.9455)	-14.5817 (0.1028)	5	300	50	0.1	100	0.01
2.2487 (0.0613)	0.0693 (0.0039)	-15.1325 (0.9511)	-14.1457 (0.1016)	5	30	50	0.1	200	0.01
2.2345 (0.0742)	0.0695 (0.0035)	-15.1317 (0.9504)	-14.1398 (0.1041)	5	150	50	0.1	200	0.01
2.2553 (0.0877)	0.0695 (0.0041)	-15.1317 (0.9504)	-14.1398 (0.1041)	5	300	50	0.1	200	0.01
2.3264 (0.0653)	0.0684 (0.0035)	-15.1789 (0.9617)	-13.4934 (0.0761)	30	30	20	0.1	100	0.001
3.9905 (0.1549)	0.0737 (0.004)	-15.1981 (0.9401)	-10.1359 (0.0666)	30	150	20	0.1	100	0.001
5.347 (0.2206)	0.0752 (0.0045)	-15.2744 (0.963)	-8.608 (0.1362)	30	300	20	0.1	100	0.001
2.8465 (0.1234)	0.0732 (0.0036)	-15.1175 (0.9657)	-12.5021 (0.0749)	30	30	20	0.1	200	0.001
6.0726 (0.2917)	0.0836 (0.0041)	-15.275 (0.9454)	-8.0977 (0.0686)	30	150	20	0.1	200	0.001
8.5148 (0.3499)	0.0954 (0.0178)	-15.3958 (0.9623)	-6.2598 (0.1536)	30	300	20	0.1	200	0.001
2.3558 (0.062)	0.0695 (0.004)	-15.1785 (0.9575)	-13.4933 (0.0801)	30	30	20	0.1	100	0.01
3.961 (0.151)	0.0736 (0.0046)	-15.1963 (0.9539)	-10.1341 (0.0674)	30	150	20	0.1	100	0.01
5.3514 (0.2251)	0.0755 (0.0043)	-15.2638 (0.9455)	-8.6456 (0.1118)	30	300	20	0.1	100	0.01
2.881 (0.1526)	0.0737 (0.0036)	-15.1227 (0.9577)	-12.4999 (0.0786)	30	30	20	0.1	200	0.01
5.9665 (0.3088)	0.0821 (0.0039)	-15.2717 (0.9577)	-8.096 (0.0641)	30	150	20	0.1	200	0.01
8.5427 (0.298)	0.0939 (0.0039)	-15.3857 (0.9519)	-6.2737 (0.1314)	30	300	20	0.1	200	0.01

2.1823 (0.0908)	0.07 (0.0048)	-15.1498 (0.9541)	-13.7645 (0.0874)	30	30	50	0.1	100	0.001
3.4348 (0.1396)	0.0735 (0.0035)	-15.1568 (0.9575)	-10.8989 (0.0978)	30	150	50	0.1	100	0.001
4.325 (0.2753)	0.0761 (0.0043)	-15.2019 (0.9571)	-9.9924 (0.1338)	30	300	50	0.1	100	0.001
2.6893 (0.1033)	0.0747 (0.004)	-15.1023 (0.9545)	-12.8481 (0.0817)	30	30	50	0.1	200	0.001
5.198 (0.2116)	0.0857 (0.0046)	-15.256 (0.9566)	-8.9089 (0.0981)	30	150	50	0.1	200	0.001
6.5284 (0.3461)	0.0955 (0.0051)	-15.324 (0.9627)	-7.8639 (0.1803)	30	300	50	0.1	200	0.001
2.2302 (0.0852)	0.0696 (0.0036)	-15.1492 (0.9513)	-13.7661 (0.086)	30	30	50	0.1	100	0.01
3.5173 (0.1067)	0.0744 (0.0041)	-15.1577 (0.9622)	-10.9058 (0.105)	30	150	50	0.1	100	0.01
4.3027 (0.1731)	0.0766 (0.0042)	-15.2031 (0.9686)	-9.9759 (0.1317)	30	300	50	0.1	100	0.01
2.714 (0.1056)	0.0738 (0.0037)	-15.1002 (0.9508)	-12.846 (0.0776)	30	30	50	0.1	200	0.01
5.1811 (0.1893)	0.086 (0.0044)	-15.2625 (0.9642)	-8.928 (0.1111)	30	150	50	0.1	200	0.01
6.572 (0.3361)	0.096 (0.0054)	-15.3266 (0.9652)	-7.8616 (0.1645)	30	300	50	0.1	200	0.01

Supplementary Table 3: Operative model hyperparameter tuning results.

Mean fit time (SD)	Mean score time (SD)	Mean test score (SD)	Mean train score (SD)	Max depth	Leaves (n)	Minimum child samples	Learning rate	Estimators (n)	Min split gain
2.3108 (0.0779)	0.0682 (0.0036)	-55.6488 (1.7267)	-49.1192 (0.167)	-1	30	20	0.1	100	0.001
3.8859 (0.175)	0.0722 (0.0034)	-55.4826 (1.6812)	-34.9245 (0.1503)	-1	150	20	0.1	100	0.001
5.8271 (0.2448)	0.0766 (0.0037)	-55.834 (1.6489)	-26.1494 (0.1407)	-1	300	20	0.1	100	0.001
2.8355 (0.1401)	0.0728 (0.004)	-55.2832 (1.7009)	-45.2377 (0.157)	-1	30	20	0.1	200	0.001
5.8935 (0.2636)	0.0808 (0.0041)	-55.5134 (1.63)	-27.0502 (0.1635)	-1	150	20	0.1	200	0.001
9.9405 (0.5061)	0.095 (0.0052)	-55.9795 (1.6459)	-16.9771 (0.1279)	-1	300	20	0.1	200	0.001
2.282 (0.0612)	0.0684 (0.0039)	-55.6433 (1.7419)	-49.1186 (0.1753)	-1	30	20	0.1	100	0.01
3.9224 (0.1854)	0.0734 (0.0034)	-55.5241 (1.6857)	-34.9299 (0.1596)	-1	150	20	0.1	100	0.01
5.8197 (0.2696)	0.0763 (0.0042)	-55.8884 (1.6552)	-26.1599 (0.1336)	-1	300	20	0.1	100	0.01
2.778 (0.0976)	0.0716 (0.0039)	-55.2736 (1.7096)	-45.2517 (0.1897)	-1	30	20	0.1	200	0.01
5.7359 (0.2025)	0.0804 (0.0039)	-55.5685 (1.6569)	-27.0429 (0.1989)	-1	150	20	0.1	200	0.01
9.4334 (0.3419)	0.093 (0.0034)	-56.0008 (1.645)	-16.9945 (0.1237)	-1	300	20	0.1	200	0.01
2.1545 (0.0915)	0.0678 (0.003)	-55.6314 (1.7875)	-50.1696 (0.1872)	-1	30	50	0.1	100	0.001
3.3866 (0.1258)	0.0733 (0.0043)	-55.5383 (1.7604)	-37.6248 (0.1539)	-1	150	50	0.1	100	0.001
4.8527 (0.1778)	0.0789 (0.0044)	-55.7922 (1.6648)	-30.7874 (0.1776)	-1	300	50	0.1	100	0.001
2.595 (0.0999)	0.0728 (0.0035)	-55.2075 (1.7594)	-46.6348 (0.1941)	-1	30	50	0.1	200	0.001
4.9632 (0.1909)	0.0857 (0.0047)	-55.6843 (1.7542)	-29.2798 (0.1761)	-1	150	50	0.1	200	0.001
7.9289 (0.213)	0.1101 (0.0065)	-56.117 (1.651)	-20.2591 (0.2261)	-1	300	50	0.1	200	0.001
2.1555 (0.0507)	0.0687 (0.0035)	-55.6281 (1.7768)	-50.1764 (0.1994)	-1	30	50	0.1	100	0.01
3.4184 (0.1161)	0.0745 (0.0037)	-55.5576 (1.7528)	-37.5966 (0.1716)	-1	150	50	0.1	100	0.01

4.8815 (0.185)	0.079 (0.0041)	-55.8122 (1.6548)	-30.7891 (0.1899)	-1	300	50	0.1	100	0.01
2.594 (0.1101)	0.073 (0.0033)	-55.2244 (1.7536)	-46.6315 (0.1849)	-1	30	50	0.1	200	0.01
5.0203 (0.217)	0.0859 (0.0048)	-55.6867 (1.7487)	-29.2622 (0.165)	-1	150	50	0.1	200	0.01
7.9734 (0.2798)	0.108 (0.0049)	-56.1349 (1.6364)	-20.2908 (0.2256)	-1	300	50	0.1	200	0.01
2.0458 (0.0702)	0.0664 (0.0037)	-56.4209 (1.7905)	-52.6815 (0.2201)	5	30	20	0.1	100	0.001
2.0589 (0.0854)	0.0661 (0.0035)	-56.4155 (1.7869)	-52.6326 (0.2132)	5	150	20	0.1	100	0.001
2.0497 (0.0765)	0.0674 (0.0062)	-56.4155 (1.7869)	-52.6326 (0.2132)	5	300	20	0.1	100	0.001
2.3573 (0.0686)	0.0699 (0.0041)	-55.7348 (1.7338)	-50.4217 (0.2229)	5	30	20	0.1	200	0.001
2.3558 (0.0917)	0.0692 (0.0029)	-55.7402 (1.744)	-50.3712 (0.2099)	5	150	20	0.1	200	0.001
2.3887 (0.1018)	0.0693 (0.0037)	-55.7402 (1.744)	-50.3712 (0.2099)	5	300	20	0.1	200	0.001
2.0495 (0.0708)	0.0661 (0.0035)	-56.4195 (1.7987)	-52.6849 (0.2084)	5	30	20	0.1	100	0.01
2.0421 (0.0543)	0.066 (0.0034)	-56.4169 (1.7833)	-52.6308 (0.2135)	5	150	20	0.1	100	0.01
2.0253 (0.0643)	0.0659 (0.0034)	-56.4169 (1.7833)	-52.6308 (0.2135)	5	300	20	0.1	100	0.01
2.3496 (0.0667)	0.0699 (0.0037)	-55.738 (1.7407)	-50.4182 (0.214)	5	30	20	0.1	200	0.01
2.3562 (0.0927)	0.0694 (0.0044)	-55.739 (1.7423)	-50.381 (0.2054)	5	150	20	0.1	200	0.01
2.3644 (0.0875)	0.0697 (0.0041)	-55.739 (1.7423)	-50.381 (0.2054)	5	300	20	0.1	200	0.01
1.9498 (0.0489)	0.0668 (0.0034)	-56.4954 (1.784)	-53.6608 (0.1882)	5	30	50	0.1	100	0.001
1.9568 (0.055)	0.0664 (0.0035)	-56.4822 (1.7966)	-53.6558 (0.2036)	5	150	50	0.1	100	0.001
1.9667 (0.0996)	0.066 (0.0036)	-56.4822 (1.7966)	-53.6558 (0.2036)	5	300	50	0.1	100	0.001
2.2166 (0.0846)	0.0689 (0.0033)	-55.7953 (1.7513)	-51.7287 (0.1844)	5	30	50	0.1	200	0.001
2.2206 (0.0808)	0.0693 (0.0032)	-55.7686 (1.7685)	-51.7102 (0.2036)	5	150	50	0.1	200	0.001
2.2337 (0.0883)	0.0694 (0.0041)	-55.7686 (1.7685)	-51.7102 (0.2036)	5	300	50	0.1	200	0.001
1.9376 (0.0335)	0.0663 (0.0035)	-56.4915 (1.7821)	-53.6572 (0.1897)	5	30	50	0.1	100	0.01

1.9385 (0.0342)	0.0659 (0.0036)	-56.4783 (1.7969)	-53.6616 (0.2043)	5	150	50	0.1	100	0.01
1.9276 (0.0636)	0.0658 (0.0033)	-56.4783 (1.7969)	-53.6616 (0.2043)	5	300	50	0.1	100	0.01
2.2361 (0.1007)	0.07 (0.0036)	-55.7931 (1.7478)	-51.7319 (0.1827)	5	30	50	0.1	200	0.01
2.2012 (0.0685)	0.0698 (0.0036)	-55.7665 (1.775)	-51.7178 (0.1995)	5	150	50	0.1	200	0.01
2.2374 (0.1131)	0.0695 (0.0032)	-55.7665 (1.775)	-51.7178 (0.1995)	5	300	50	0.1	200	0.01
2.2826 (0.0571)	0.0685 (0.0041)	-55.6488 (1.7267)	-49.1192 (0.167)	30	30	20	0.1	100	0.001
3.8379 (0.1474)	0.0724 (0.0032)	-55.5193 (1.7198)	-35.1342 (0.1448)	30	150	20	0.1	100	0.001
5.3515 (0.2072)	0.0744 (0.0027)	-55.7614 (1.7041)	-28.0574 (0.2424)	30	300	20	0.1	100	0.001
2.8026 (0.1177)	0.0721 (0.004)	-55.2832 (1.7009)	-45.2377 (0.157)	30	30	20	0.1	200	0.001
5.7403 (0.1998)	0.0805 (0.0037)	-55.5721 (1.7048)	-27.3035 (0.167)	30	150	20	0.1	200	0.001
8.7082 (0.2864)	0.0925 (0.0037)	-55.8766 (1.6886)	-18.7699 (0.1765)	30	300	20	0.1	200	0.001
2.2767 (0.101)	0.0678 (0.0036)	-55.6433 (1.7419)	-49.1186 (0.1753)	30	30	20	0.1	100	0.01
3.7941 (0.1782)	0.0721 (0.004)	-55.5242 (1.6943)	-35.1262 (0.1497)	30	150	20	0.1	100	0.01
5.2529 (0.2175)	0.0754 (0.0044)	-55.8457 (1.6615)	-28.064 (0.2756)	30	300	20	0.1	100	0.01
2.7735 (0.0949)	0.073 (0.0034)	-55.2736 (1.7096)	-45.2517 (0.1897)	30	30	20	0.1	200	0.01
5.8377 (0.2362)	0.0814 (0.0042)	-55.5709 (1.6825)	-27.306 (0.1592)	30	150	20	0.1	200	0.01
8.8 (0.3293)	0.0935 (0.0049)	-55.9799 (1.659)	-18.7724 (0.2187)	30	300	20	0.1	200	0.01
2.1629 (0.0819)	0.0692 (0.0035)	-55.6314 (1.7875)	-50.1696 (0.1872)	30	30	50	0.1	100	0.001
3.3952 (0.1439)	0.0734 (0.0047)	-55.4779 (1.7237)	-38.1323 (0.2267)	30	150	50	0.1	100	0.001
4.1681 (0.1417)	0.0757 (0.0039)	-55.5927 (1.711)	-34.1709 (0.3608)	30	300	50	0.1	100	0.001
2.605 (0.1334)	0.0739 (0.0051)	-55.2075 (1.7594)	-46.6348 (0.1941)	30	30	50	0.1	200	0.001
4.9246 (0.1785)	0.0848 (0.0038)	-55.6088 (1.7134)	-29.7084 (0.2019)	30	150	50	0.1	200	0.001
6.5788 (0.2766)	0.0958 (0.0039)	-55.81 (1.6812)	-24.097 (0.4404)	30	300	50	0.1	200	0.001

2.1814 (0.1664)	0.0688 (0.0038)	-55.6281 (1.7768)	-50.1764 (0.1994)	30	30	50	0.1	100	0.01
3.3179 (0.1348)	0.0735 (0.0031)	-55.4867 (1.7578)	-38.1251 (0.2184)	30	150	50	0.1	100	0.01
4.2285 (0.1778)	0.0752 (0.0035)	-55.6639 (1.6932)	-34.2116 (0.4185)	30	300	50	0.1	100	0.01
2.5802 (0.0787)	0.0732 (0.0032)	-55.2244 (1.7536)	-46.6315 (0.1849)	30	30	50	0.1	200	0.01
5.0113 (0.2367)	0.0846 (0.004)	-55.6175 (1.7425)	-29.7443 (0.2043)	30	150	50	0.1	200	0.01
6.6522 (0.2925)	0.0961 (0.0044)	-55.9268 (1.6233)	-24.1444 (0.4064)	30	300	50	0.1	200	0.01

Supplementary Table 4: Post-procedure model hyperparameter tuning results

Mean fit time (SD)	Mean score time (SD)	Mean test score (SD)	Mean train score (SD)	Max depth	Leaves (n)	Minimum child samples	Learning rate	Estimators (n)	min split gain
2.2746 (0.1033)	0.0689 (0.0037)	-11.9669 (0.8567)	-10.0215 (0.0663)	-1	30	20	0.1	100	0.001
3.9636 (0.2133)	0.0724 (0.0035)	-12.1203 (0.8559)	-7.2051 (0.049)	-1	150	20	0.1	100	0.001
5.9942 (0.257)	0.0768 (0.0034)	-12.2097 (0.8547)	-5.7967 (0.0565)	-1	300	20	0.1	100	0.001
2.8626 (0.139)	0.0725 (0.0036)	-12.0109 (0.8549)	-9.2172 (0.0592)	-1	30	20	0.1	200	0.001
5.9721 (0.2505)	0.0829 (0.0043)	-12.2017 (0.8523)	-5.6708 (0.0398)	-1	150	20	0.1	200	0.001
10.1796 (0.5153)	0.1015 (0.0061)	-12.324 (0.8438)	-3.838 (0.0341)	-1	300	20	0.1	200	0.001
2.302 (0.0589)	0.0689 (0.0029)	-11.9693 (0.8594)	-10.0178 (0.067)	-1	30	20	0.1	100	0.01
4.0611 (0.2334)	0.0735 (0.004)	-12.1284 (0.849)	-7.2064 (0.0529)	-1	150	20	0.1	100	0.01
5.8808 (0.214)	0.0777 (0.0038)	-12.2068 (0.8584)	-5.8011 (0.0567)	-1	300	20	0.1	100	0.01
2.7549 (0.111)	0.072 (0.004)	-12.0092 (0.8552)	-9.2197 (0.056)	-1	30	20	0.1	200	0.01
5.7646 (0.1652)	0.081 (0.0041)	-12.2151 (0.8522)	-5.6705 (0.0495)	-1	150	20	0.1	200	0.01
9.9208 (0.361)	0.0985 (0.0047)	-12.3124 (0.8542)	-3.8476 (0.0416)	-1	300	20	0.1	200	0.01
2.1165 (0.1037)	0.0681 (0.0031)	-11.9281 (0.8714)	-10.4161 (0.0752)	-1	30	50	0.1	100	0.001
3.3877 (0.1251)	0.0737 (0.0031)	-12.0542 (0.8633)	-8.1121 (0.0713)	-1	150	50	0.1	100	0.001
5.1713 (0.2387)	0.0808 (0.0049)	-12.1144 (0.8495)	-7.2083 (0.0808)	-1	300	50	0.1	100	0.001
2.6447 (0.1052)	0.074 (0.0044)	-11.9724 (0.8706)	-9.6975 (0.0669)	-1	30	50	0.1	200	0.001
5.2618 (0.2021)	0.0868 (0.0046)	-12.1929 (0.8523)	-6.3587 (0.0514)	-1	150	50	0.1	200	0.001
8.3374 (0.298)	0.1141 (0.0052)	-12.2801 (0.8374)	-5.0225 (0.0663)	-1	300	50	0.1	200	0.001
2.1307 (0.0602)	0.068 (0.0031)	-11.926 (0.8719)	-10.4139 (0.0766)	-1	30	50	0.1	100	0.01
3.5443 (0.1394)	0.0743 (0.0041)	-12.0535 (0.8591)	-8.1161 (0.0702)	-1	150	50	0.1	100	0.01

5.0815 (0.2832)	0.0805 (0.0035)	-12.1139 (0.8425)	-7.2104 (0.0813)	-1	300	50	0.1	100	0.01
2.6233 (0.1129)	0.0727 (0.0042)	-11.9663 (0.8686)	-9.6931 (0.0749)	-1	30	50	0.1	200	0.01
5.1777 (0.2299)	0.0866 (0.0038)	-12.1918 (0.8441)	-6.3629 (0.0492)	-1	150	50	0.1	200	0.01
8.4548 (0.3976)	0.1136 (0.0066)	-12.2802 (0.8314)	-5.0224 (0.0685)	-1	300	50	0.1	200	0.01
2.0514 (0.1039)	0.0663 (0.0028)	-11.9143 (0.872)	-10.9722 (0.0809)	5	30	20	0.1	100	0.001
2.0637 (0.0886)	0.0664 (0.0035)	-11.9167 (0.8706)	-10.9739 (0.0801)	5	150	20	0.1	100	0.001
2.0988 (0.0812)	0.068 (0.0047)	-11.9167 (0.8706)	-10.9739 (0.0801)	5	300	20	0.1	100	0.001
2.3597 (0.0934)	0.0698 (0.0035)	-11.9274 (0.8689)	-10.5859 (0.0802)	5	30	20	0.1	200	0.001
2.3608 (0.1042)	0.0696 (0.0037)	-11.9284 (0.8703)	-10.5851 (0.0765)	5	150	20	0.1	200	0.001
2.3661 (0.1018)	0.0697 (0.0041)	-11.9284 (0.8703)	-10.5851 (0.0765)	5	300	20	0.1	200	0.001
2.0493 (0.0754)	0.0677 (0.004)	-11.9132 (0.8736)	-10.9748 (0.0818)	5	30	20	0.1	100	0.01
2.0715 (0.0729)	0.0672 (0.0049)	-11.9138 (0.8696)	-10.9731 (0.0802)	5	150	20	0.1	100	0.01
2.0707 (0.1034)	0.0668 (0.0033)	-11.9138 (0.8696)	-10.9731 (0.0802)	5	300	20	0.1	100	0.01
2.395 (0.1135)	0.0701 (0.0036)	-11.9231 (0.8712)	-10.5851 (0.0775)	5	30	20	0.1	200	0.01
2.4058 (0.1053)	0.0708 (0.0039)	-11.9304 (0.8691)	-10.5822 (0.0812)	5	150	20	0.1	200	0.01
2.3878 (0.0942)	0.0696 (0.003)	-11.9304 (0.8691)	-10.5822 (0.0812)	5	300	20	0.1	200	0.01
1.9749 (0.1327)	0.0658 (0.0033)	-11.9025 (0.8811)	-11.184 (0.0903)	5	30	50	0.1	100	0.001
1.9393 (0.0601)	0.0664 (0.0034)	-11.9026 (0.8849)	-11.1825 (0.0912)	5	150	50	0.1	100	0.001
1.9494 (0.07)	0.0669 (0.0039)	-11.9026 (0.8849)	-11.1825 (0.0912)	5	300	50	0.1	100	0.001
2.2689 (0.0854)	0.0698 (0.0037)	-11.9088 (0.8779)	-10.8413 (0.0868)	5	30	50	0.1	200	0.001
2.2887 (0.0774)	0.0702 (0.0036)	-11.9096 (0.8806)	-10.8394 (0.0836)	5	150	50	0.1	200	0.001
2.2764 (0.1044)	0.0699 (0.0036)	-11.9096 (0.8806)	-10.8394 (0.0836)	5	300	50	0.1	200	0.001
1.9345 (0.0575)	0.0658 (0.0035)	-11.9012 (0.8816)	-11.183 (0.0888)	5	30	50	0.1	100	0.01

1.9663 (0.0543)	0.0669 (0.0033)	-11.9031 (0.8812)	-11.1833 (0.0907)	5	150	50	0.1	100	0.01
1.9802 (0.0685)	0.0664 (0.0038)	-11.9031 (0.8812)	-11.1833 (0.0907)	5	300	50	0.1	100	0.01
2.2776 (0.0909)	0.0703 (0.0046)	-11.9061 (0.8755)	-10.8411 (0.0844)	5	30	50	0.1	200	0.01
2.3004 (0.127)	0.07 (0.0039)	-11.9078 (0.8792)	-10.8408 (0.0854)	5	150	50	0.1	200	0.01
2.2169 (0.061)	0.0693 (0.0036)	-11.9078 (0.8792)	-10.8408 (0.0854)	5	300	50	0.1	200	0.01
2.2523 (0.0586)	0.0683 (0.0031)	-11.9669 (0.8567)	-10.0215 (0.0663)	30	30	20	0.1	100	0.001
3.9207 (0.2089)	0.073 (0.0036)	-12.1215 (0.8603)	-7.227 (0.0526)	30	150	20	0.1	100	0.001
5.6493 (0.2117)	0.0771 (0.0049)	-12.1978 (0.8372)	-5.9955 (0.0512)	30	300	20	0.1	100	0.001
2.7944 (0.1317)	0.0719 (0.0037)	-12.0109 (0.8549)	-9.2172 (0.0592)	30	30	20	0.1	200	0.001
5.94 (0.222)	0.0821 (0.0038)	-12.2093 (0.8505)	-5.6942 (0.0435)	30	150	20	0.1	200	0.001
9.6801 (0.407)	0.0996 (0.0046)	-12.3129 (0.8277)	-4.0059 (0.0377)	30	300	20	0.1	200	0.001
2.2574 (0.0774)	0.0686 (0.0036)	-11.9693 (0.8594)	-10.0178 (0.067)	30	30	20	0.1	100	0.01
3.9046 (0.1561)	0.0728 (0.004)	-12.1263 (0.8515)	-7.2347 (0.0499)	30	150	20	0.1	100	0.01
5.7285 (0.2266)	0.0764 (0.004)	-12.2153 (0.8488)	-6.0027 (0.0594)	30	300	20	0.1	100	0.01
2.8709 (0.3199)	0.0729 (0.0046)	-12.0092 (0.8552)	-9.2197 (0.056)	30	30	20	0.1	200	0.01
6.2151 (0.2565)	0.0841 (0.0048)	-12.2067 (0.8447)	-5.6968 (0.045)	30	150	20	0.1	200	0.01
9.7531 (0.4199)	0.099 (0.0037)	-12.3348 (0.8406)	-4.0084 (0.0439)	30	300	20	0.1	200	0.01
2.1674 (0.0877)	0.0699 (0.0038)	-11.9281 (0.8714)	-10.4161 (0.0752)	30	30	50	0.1	100	0.001
3.5221 (0.1504)	0.0752 (0.004)	-12.0562 (0.8514)	-8.1489 (0.0736)	30	150	50	0.1	100	0.001
4.5984 (0.1946)	0.0765 (0.0035)	-12.0995 (0.8646)	-7.569 (0.0917)	30	300	50	0.1	100	0.001
2.6909 (0.1157)	0.0741 (0.0046)	-11.9724 (0.8706)	-9.6975 (0.0669)	30	30	50	0.1	200	0.001
5.3349 (0.1974)	0.0871 (0.0042)	-12.1913 (0.8362)	-6.3937 (0.0553)	30	150	50	0.1	200	0.001
7.3165 (0.3647)	0.1013 (0.0051)	-12.258 (0.8493)	-5.4877 (0.0795)	30	300	50	0.1	200	0.001

2.1244 (0.061)	0.0692 (0.0036)	-11.926 (0.8719)	-10.4139 (0.0766)	30	30	50	0.1	100	0.01
3.4074 (0.1439)	0.0739 (0.0039)	-12.0538 (0.8567)	-8.1514 (0.0651)	30	150	50	0.1	100	0.01
4.4178 (0.2115)	0.0769 (0.004)	-12.0929 (0.8489)	-7.5632 (0.0861)	30	300	50	0.1	100	0.01
2.5998 (0.13)	0.0736 (0.0043)	-11.9663 (0.8686)	-9.6931 (0.0749)	30	30	50	0.1	200	0.01
5.0846 (0.236)	0.0857 (0.0034)	-12.1924 (0.8433)	-6.3909 (0.0478)	30	150	50	0.1	200	0.01
6.9482 (0.1852)	0.0995 (0.0046)	-12.2468 (0.8425)	-5.4789 (0.0729)	30	300	50	0.1	200	0.01

Supplementary Table 5: Continuous Model Features by Training and Holdout Sets

Variable	Train Median [IQR]	Holdout Median [IQR]
Body Mass Index	28.94 [84.00-11.00]	28.84 [77.00-12.00]
Claims Frailty Index	0.12 [0.40-0.05]	0.13 [0.42-0.05]
Comorbidity Count	2.76 [33.00-0.00]	2.99 [27.00-0.00]
Driving Distance (Mi)	46.33 [4600.30-1.10]	46.76 [2916.80-1.10]
Total Elixhauser Comorbidities	2.59 [21.00-0.00]	2.75 [23.00-0.00]
In Room to Out Room Time (min)	174.39 [1456.20-8.00]	171.28 [1009.50-10.10]
Operative Time (min)	109.28 [981.60-1.10]	108.21 [711.60-1.70]
Post-procedure Time (min)	19.64 [144.30-0.80]	19.22 [111.30-1.50]
Pre-procedure Time (min)	45.47 [363.40-2.00]	43.85 [245.00-2.90]
Mean Arterial Pressure (most recent)	93.30 [160.70-0.00]	92.96 [148.30-25.30]
Number of Panels	1.08 [5.00-1.00]	1.08 [4.00-1.00]
Total Allergies	1.45 [60.00-0.00]	1.50 [61.00-0.00]
Previous Critical Care Count	0.76 [90.00-0.00]	0.65 [82.00-0.00]
Previous Inpatient Count	2.62 [90.00-0.00]	2.69 [90.00-0.00]
Provider 90-day Operative Volume	467.92 [2811.00-0.00]	489.75 [2859.00-0.00]
Provider OR Constancy	67.76 [569.00-1.00]	93.87 [566.00-1.00]
Pulse (most recent)	76.94 [231.00-0.00]	76.79 [226.00-0.00]
Respiratory Rate (most recent)	17.11 [60.00-0.00]	17.12 [60.00-0.00]
Below Poverty Line (%)	5.78 [39.98-0.00]	5.69 [66.67-0.00]
Gini Index	0.45 [0.71-0.02]	0.45 [0.71-0.07]
Median Income by Race	81813.95 [250001-2499]	81669.36 [250001.00-2499.00]
Median Rent	1248.96 [3501-377]	1243.85 [3501.00-354.00]
Medicaid Coverage (%)	15.21 [67.58-0.00]	15.23 [60.03-0.00]
No Vehicle Access (%)	13.45 [85.47-0.00]	13.48 [82.52-0.00]
Limited English Proficiency (%)	2.46 [41.40-0.00]	2.45 [41.40-0.00]
Unemployment Rate (%)	6.10 [74.47-0.00]	6.10 [23.36-0.00]
Uninsured Rate (%)	5.35 [55.51-0.00]	5.31 [55.51-0.00]
Vacant Housing (%)	9.40 [96.04-0.00]	9.24 [96.20-0.00]

Supplementary Table 6: Case Distribution by Surgical Service for the Training and Holdout Sets

Surgical Service	Train (n, %)	Holdout (n, %)
Breast Surgery	328 (0.7%)	246 (2.8%)
Cardiac Surgery	3611 (7.7%)	613 (7.0%)
Cardiovascular Surgery	261 (0.6%)	59 (0.7%)
Colorectal Surgery	2140 (4.6%)	353 (4.0%)
Endocrine & Oncologic Surgery	5178 (11.1%)	771 (8.8%)
Gastrointestinal Surgery	2948 (6.3%)	551 (6.3%)
Gynecology	3793 (8.1%)	679 (7.8%)
Neurosurgery	4241 (9.1%)	815 (9.3%)
Oral & Maxillofacial Surgery	1029 (2.2%)	214 (2.5%)
Otolaryngology	5006 (10.7%)	921 (10.6%)
Otolaryngology - Head & Neck Surgery	933 (2.0%)	153 (1.8%)
Orthopedic Surgery	517 (1.1%)	90 (1.0%)
Plastic Surgery	3598 (7.7%)	692 (7.9%)
Thoracic Surgery	1432 (3.1%)	332 (3.8%)
Transplant Surgery	1063 (2.3%)	193 (2.2%)
Trauma Surgery	2193 (4.7%)	398 (4.6%)
Urology	6136 (13.1%)	1196 (13.7%)
Vascular Surgery	2222 (4.8%)	445 (5.1%)
Other	89 + 4 + 14 + 7 +18 + 2 + 4	0+0+3+0+3+1+0
Total	46767 (100.0%)	8728 (100.0%)
